# Identifying active vascular microcalcification by ^18^F-sodium fluoride positron emission tomography

**DOI:** 10.1038/ncomms8495

**Published:** 2015-07-07

**Authors:** Agnese Irkle, Alex T. Vesey, David Y. Lewis, Jeremy N. Skepper, Joseph L. E. Bird, Marc R. Dweck, Francis R. Joshi, Ferdia A. Gallagher, Elizabeth A. Warburton, Martin R. Bennett, Kevin M. Brindle, David E. Newby, James H. Rudd, Anthony P. Davenport

**Affiliations:** 1 Division of Experimental Medicine & Immunotherapeutics (EMIT), Department of Medicine, University of Cambridge, Cambridge, CB2 0QQ, UK.; 2 Centre for Cardiovascular Science, University of Edinburgh, Edinburgh, EH16 4TJ, UK.; 3 Cancer Research UK Cambridge Institute, University of Cambridge, Li Ka Shing Centre, Cambridge, CB2 0RE, UK.; 4 Department of Physiology, Development and Neuroscience, Multi-Imaging Centre, University of Cambridge, Cambridge, CB2 3EG, UK.; 5 Division of Cardiovascular Medicine, University of Cambridge, Cambridge, CB2 0QQ, UK.; 6 Department of Radiology, Box 218 Level 5, University of Cambridge, Cambridge, CB2 0QQ, UK.; 7 Department of Clinical Neurosciences, University of Cambridge, Cambridge, CB2 0QQ, UK.

## Abstract

Vascular calcification is a complex biological process that is a hallmark of atherosclerosis. While macrocalcification confers plaque stability, microcalcification is a key feature of high-risk atheroma and is associated with increased morbidity and mortality. Positron emission tomography and X-ray computed tomography (PET/CT) imaging of atherosclerosis using ^18^F-sodium fluoride (^18^F-NaF) has the potential to identify pathologically high-risk nascent microcalcification. However, the precise molecular mechanism of ^18^F-NaF vascular uptake is still unknown. Here we use electron microscopy, autoradiography, histology and preclinical and clinical PET/CT to analyse ^18^F-NaF binding. We show that ^18^F-NaF adsorbs to calcified deposits within plaque with high affinity and is selective and specific. ^18^F-NaF PET/CT imaging can distinguish between areas of macro- and microcalcification. This is the only currently available clinical imaging platform that can non-invasively detect microcalcification in active unstable atherosclerosis. The use of ^18^F-NaF may foster new approaches to developing treatments for vascular calcification.

## Abstract (web summary)

Atherosclerotic plaques with macrocalcification are stable, whereas microcalcification is a key feature of rupture-prone plaques. Here the authors show that ^18^F-NaF PET/CT imaging can distinguish between macro- and microcalcification providing a potential, non-invasive imaging technique to identify patients with high-risk atheroma.

## Graphical abstract

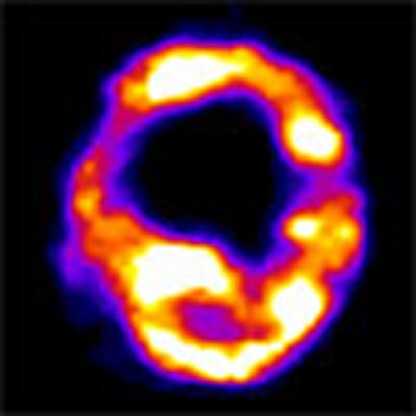

Vascular calcification is a complex pathological process pathognomonic of atherosclerosis^1^,^2^,^3^,^4^. Currently, the extent of macroscopic calcification can be determined by X-ray computed tomography (CT) imaging and this can be used clinically to predict cardiovascular morbidity and mortality^5^,^6^,^7^.

Vessel mineralization is first marked by the appearance of calcified micro nodules which grow and coalesce into much larger macroscopic deposits^8^. The genesis of these micro nodules is principally mediated by a coordinated cellular pathway that shares similarities to active skeletal osteogenesis but may also involve a more passive process where a combination of high local concentrations of phosphates and phosphatidylserines from necrotic cells and an absence of calcification inhibitors results in the precipitation of calcium phosphate particles. It is likely that much of the mineral present is made up of hydroxyapatite, a crystalline form of calcium orthophosphate^9^,^10^. Although there is no universal size convention, emerging consensus categorizes micro- and macrocalcification based on nodules of <50 and ≥50 μm, respectively^11^. This distinction is important because although macrocalcification imparts plaque stability, microcalcification heralds the onset of vessel mineralization triggered by cell death and inflammation^8^,^12^ and may itself be implicated in the aetiology of plaque rupture and major adverse cardiovascular events^13^,^14^. Detection of microcalcification is not possible with clinical CT systems that can only identify large areas of macrocalcification—∼200–500 μm in diameter^10^,^15^,^16^,^17^. Furthermore, the rapid progression of existing vascular calcium is driven by on-going microcalcification, carries a poor prognosis and is not amenable to current medical therapies. As a consequence, the development of techniques to identify microcalcification is a major research goal with the potential for improving patient risk stratification and outcomes.

Positron emission tomography (PET)/CT imaging of atherosclerosis using ^18^F-sodium fluoride (^18^F-NaF) has recently been reported and, for the first time, has the potential to non-invasively identify high-risk microcalcification^18^,^19^,^20^,^21^. In patients with stable coronary artery disease, ^18^F-NaF uptake correlates with CT coronary calcium scores although nearly half of the patients with very high scores have no ^18^F-NaF uptake and increased ^18^F-NaF uptake is often seen in the absence of calcification on CT. ^18^F-NaF would therefore appear to provide distinct information to CT with potentially important clinical implications^20^. Recently, we have shown that ^18^F-NaF uptake identifies culprit ruptured coronary plaques after myocardial infarction and, in carotid plaque, correlates with markers of active calcification and cell death^21^. Moreover, ^18^F-NaF appears to identify high-risk coronary lesions in stable patients indicating that it may have a role in identifying vulnerable plaques at risk of rupture and in predicting myocardial infarction. In bone imaging, ^18^F-NaF uptake involves the exchange of fluoride ions with hydroxyl groups in hydroxyapatite^22^,^23^; however, the mechanism underlying ^18^F-NaF uptake in the vasculature has not been established. This is a major limitation that must be addressed before the nascent field of vascular ^18^F-NaF PET imaging can progress.

The goal of the study was to characterize the selectivity, specificity and pharmacodynamics of ^18^F-NaF uptake in vascular tissue in the human carotid atherosclerotic plaque. First, we performed electron probe X-ray microanalysis to measure ^18^F-NaF presence directly in the areas of calcification identified by electron microscopy. Second, we studied the pharmacodynamic properties of ^18^F-NaF adsorption to vascular calcification to characterize its potential as a clinical imaging agent. We correlated the extent of ^18^F-NaF adsorption to vascular calcification and to cell-specific markers of atherosclerosis to exclude the possibility of ^18^F-NaF binding to markers of inflammation, neovascularization and smooth muscle cells as opposed to solely calcified deposits. We next compared the ability of ^18^F-NaF to penetrate carotid micro- and macrocalcifications using high-resolution phosphor-imaging autoradiography and light microscopy to understand ^18^F-NaF-binding properties to these different forms of vascular calcifications. Finally, we compared clinical ^18^F-NaF PET/CT images with μPET/μCT images of excised carotid endarterectomy specimens to develop a translational model of ^18^F-NaF vascular uptake from bedside to bench and back and understand ^18^F-NaF adsorption to vascular calcification on all three levels studied.

We propose a comprehensive translational model of ^18^F-NaF pharmacological characteristics to vascular calcification in preclinical and clinical imaging systems. We conclude that ^18^F-NaF PET/CT is the only currently available non-invasive clinical imaging platform that can detect microcalcification in active unstable human atherosclerosis.

## Results

### Electron microscopy analysis

**Figure 1:**
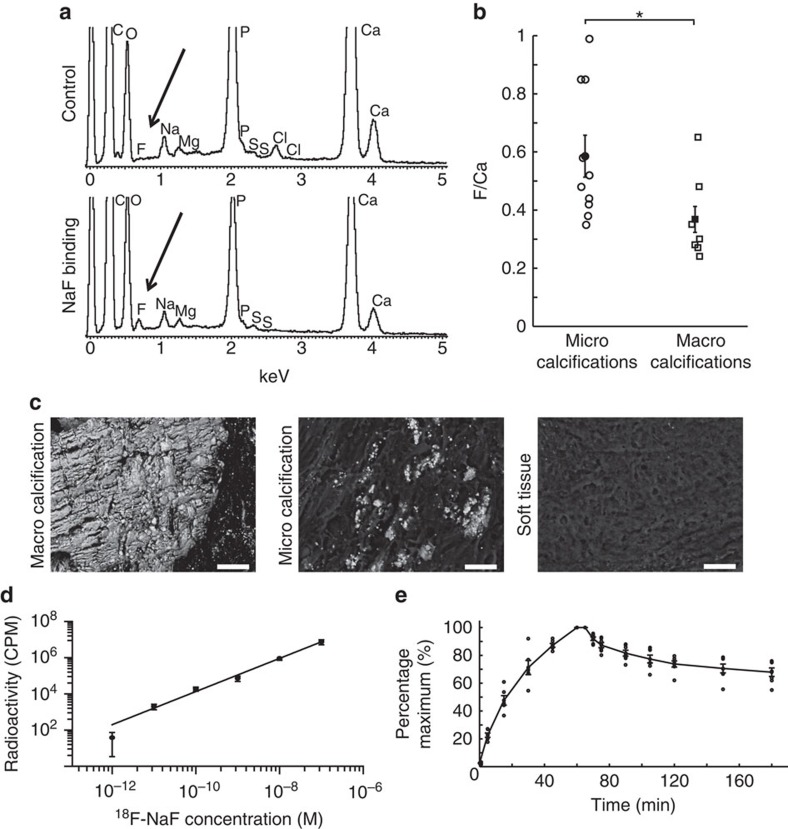
F directly co-localizes with Ca in a concentration-dependent manner. (**a**) Fluoride peak is detected only in the calcified regions of those carotids that have been exposed to NaF. (**b**) The amount of fluoride adsorbed to microcalcifications (identified visually based on the size of nodules of <50 and manual measurements made) is significantly higher than macrocalcifications (≥50 μm to several mm; F/Ca in microcalcifications 0.59±0.23, *n*=10 (circles); in macrocalcifications 0.37±0.15, *n*=7 (squares)); *P<*0.02 using an ANOVA and Tukey Kramer *post hoc* test. (**c**) Representative images of macro- and microcalcifications and the soft tissue. F presence was detected only in the calcified regions. Scale bar, 50 μm. (**d**) ^18^F-NaF binding to cryostat sections is linear over the clinically relevant concentration range from 1.0 × 10^−12^ to 1.0 × 10^−7^ (*y*=10^(0.92*log(x)+13)^, *n*=5). (**e**) There is a fast exponential association and slow exponential dissociation of ^18^F-NaF to the whole carotids over time. In all figures filled shapes show mean, error bars denote s.e.m.

We used an electron microprobe to measure directly the presence of non-radioactive fluoride within calcified and soft tissue areas of carotid endarterectomy specimens. We also used hydroxyapatite standards to confirm co-localization (see Supplementary Table 1). When tissues were preincubated with NaF, an energy-dispersive X-ray spectroscopic peak for fluoride was detectable and co-localized with regions of calcification (Fig. 1a). Microcalcifications showed greater levels of fluoride than macrocalcifications (F/Ca ratio: 0.59±0.23 (*n*=10, individual plaques) versus 0.37±0.15 (*n*=7, individual plaques), respectively, *P<*0.02, ANOVA (analysis of variance) and Tukey Kramer *post hoc* test, Fig. 1b) and no tissue fluoride was observed in the absence of calcification (Fig. 1c). Thus, at the resolution of electron microscopy, fluoride co-localized to arterial calcification, with increased levels in the regions of microcalcification.

### Pharmacodynamic and pharmacokinetic analyses

To confirm the specificity in pharmacological assays, we incubated carotid sections with a clinically relevant concentration (1 × 10^−11^ M) of ^18^F-NaF for 1 h (ref. 21). As a control for nonspecific binding, adjacent sections were incubated at the same concentration of ^18^F-NaF, but with an excess (4 × 10^−4^ M) of non-radioactive NaF. Phosphor-imaging autoradiography revealed a strong ^18^F-NaF signal with negligible nonspecific binding and no ^18^F-NaF binding in areas without calcification (Supplementary Fig. 1). Concentration–response curves were constructed by exposing carotid sections (*n*=5) to increasing concentrations of ^18^F-NaF (10^−12^–10^−7^ M) for 1 h (refs 20, 23). There was a positive linear correlation between the radioactivity and concentration of ^18^F-NaF (Fig. 1d). We then performed association and dissociation binding experiments at a clinically relevant concentration (1 × 10^−11^ M; *n*=5). There was a rapid and time-dependent exponential association and, following washing, a slow exponential dissociation from the plaque (Fig. 1e). The data were fitted to a one-site model, with an association rate constant (*k*_1_) of 4.5±0.6 × 10^9^ M^−1^ min^−1^ and a half-time for association of 14.3±1.9 min. Dissociation was slow but detectable with a dissociation rate constant of 0.0027±0.0005, min^−1^, a half-time for dissociation of 254±43 min and an affinity constant (*K*_D_) calculated from these kinetic data of 0.6 pM. The affinity constant is a measure of the strength of binding of ^18^F-NaF to areas of calcification. The very high affinity is consistent with the comparatively low concentrations of radiolabelled ^18^F-NaF required *in vitro* and *in vivo* to detect calcifications in tissues and demonstrates the sensitivity of the tracer.

We undertook dynamic PET analysis to explore the *in vivo* pharmacodynamic and pharmacokinetic properties of ^18^F-NaF in five patients scheduled to undergo carotid endarterectomy for symptomatic disease (patient demographics both for dynamic scans and static scans described later are given in Supplementary Table 2. The SUV and TBR values of static and dynamic scans in table format are given in Supplementary Table 3 and in graphical format in Supplementary Fig. 2). There was clear carotid plaque uptake and Patlak analysis showed a good model fit with ^18^F-NaF influx varying from 0.0005 to 0.007 ml cm^−3^ min^−1^ as compared with 0.05 to 0.08 ml cm^−3^ min^−1^ in the vertebral bodies (Supplementary Table 4 and Supplementary Fig. 3). No deviation of the data from the model was observed during the time course of these experiments although patients could only tolerate scanning for 75 min following tracer injection. Although very slow dissociation can be detected *in vitro*, this was too slow to detect during our imaging window *in vivo* and therefore is unlikely to be an important factor to take into account in clinical imaging. ^18^F-NaF was rapidly eliminated from the plasma and showed a typical biexponential decay in keeping with immediate redistribution and then subsequent renal elimination (Supplementary Fig. 4). At 60 min from injection, plasma activity was 4–8% of its peak activity and excellent contrast between vascular tissue and blood pool was observed.

These complementary *ex vivo* and *in vivo* data demonstrate the favourable imaging properties of ^18^F-NaF: an absence of metabolites, rapid ligand-binding site association with minimal dissociation and a rapid reduction in radiotracer background giving excellent tissue contrast resolution 1 h following its administration.

### Immunohistochemical analysis

**Figure 2:**
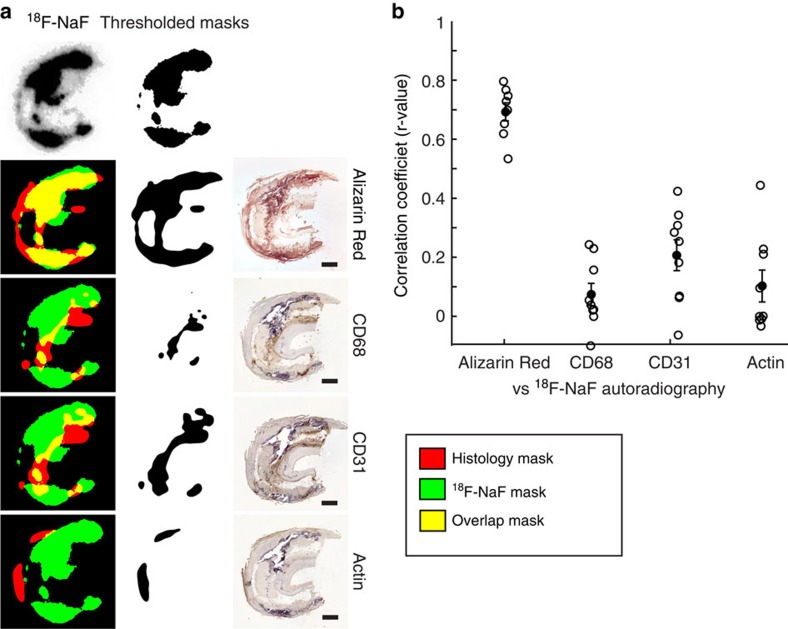
^18^F-NaF uptake correlates with calcification but none of the histological inflammatory markers. (**a**) Representative images of ^18^F-NaF autoradiography signal overlap with IHC-stained sequential sections. Green: ^18^F-NaF signal, red: histology signal, yellow: overlap. Scale bar, 1 mm. (**b**) High correlation is observed between ^18^F-NaF and Alizarin Red calcification staining, while low correlation is seen between ^18^F-NaF autoradiography and inflammatory marker IHC signals.

Vascular calcification is believed to occur in response to hypoxia, necrosis and chronic inflammation and has been linked with macrophage burden and neovascularization^3^. To test the cellular specificity of ^18^F-NaF binding, we stained sequential sections with Alizarin Red (calcification marker) and antibodies for CD68 (macrophage marker), CD31 (endothelial cell marker of neovascularization) and smooth muscle actin (smooth muscle marker; *n*=8 individual plaques for each stain). We then filtered and applied an auto-threshold to both the ^18^F-NaF radioactivity signal and each of the histological stains, and overlaid them to correlate the two signals (Fig. 2a). The only high correlation detected was between the ^18^F-NaF signal and Alizarin Red calcification stain (*r*=0.69±0.03, *n*=8) indicating that ^18^F-NaF specifically co-localizes to vascular calcification and not other upstream triggers to the calcific response (Fig. 2b).

### Penetration by autoradiography/histology and μPET/μCT

**Figure 3:**
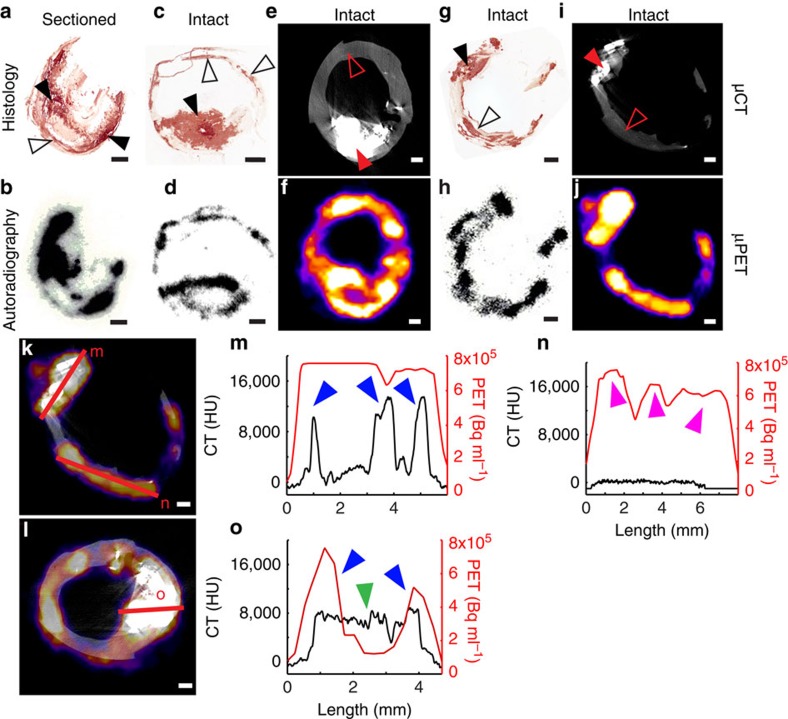
^18^F-NaF signal detection depends on the sensitivity of the detection modality. (**a**,**b**) If the carotid is sectioned first and incubated in ^18^F-NaF second, binding occurs to all macro- (closed arrowheads) and microcalcification (open arrowheads) surfaces. (**c**,**d**) However, if the carotid is incubated in ^18^F-NaF first and then sectioned, ^18^F-NaF is able to bind only to the surface level of macrocalcifications (closed arrowheads), while binding occurs to all microcalcifications (open arrowheads). (**e**,**f**) ^18^F-NaF binding solely to the surface level of macrocalcifications can also be observed in a μPET/μCT scan, if the macrocalcification size is larger than μPET resolution. (**g**,**i**) Microcalcifications that are detected with Alizarin Red histology cannot be seen in a μCT scan, due to insufficient sensitivity. (**h**,**j**) Yet, ^18^F-NaF μPET scan closely matches autoradiography signal and can detect microcalcifications. (**k**,**l**) μPET/μCT signal quantification by measuring the intensity of the ^18^F-NaF μPET signal and the μCT density measure along the same transects. (**m**) ^18^F-NaF binding can be observed throughout the three macrocalcification peaks due to macrocalcification size being smaller than μPET resolution (PET+/CT+, blue arrowheads); (**n**) Microcalcifications detected with ^18^F-NaF μPET and autoradiography (see **h**) as well as Alizarin Red histology (see **g**) but not μCT (PET+/CT−, magenta arrowheads); (**o**) If macrocalcifications are larger than μPET resolution, ^18^F-NaF binding solely around the surface level of macrocalcifications can be observed, similarly as in autoradiography (center green arrowhead PET−/CT+, blue arrowheads PET+/CT+). Scale bar, 1 mm.

We next compared areas of ^18^F-NaF uptake with tissue micro- and macrocalcification. We first sectioned carotid plaques to remove barriers to penetration and incubated with 1 × 10^−11^ M ^18^F-NaF for 1 h, followed by exposure to a phosphor-imaging screen. ^18^F-NaF uptake correlated with calcification staining (Fig. 3a, macrocalcifications: closed arrowheads, microcalcifications: open arrowheads) and was observed across all regions of the calcific deposits (Fig. 3b). In contrast, if carotids were first incubated with ^18^F-NaF and then sectioned, ^18^F-NaF binding was only detected on the outer surface layer of macrocalcific deposits with the ligand unable to penetrate deeper (Fig. 3c,d, and Supplementary Fig. 5 for additional examples). Irrespective of presectioning of tissue, ^18^F-NaF appeared to bind freely to regions of microcalcification and co-localized with histological staining for calcification (Fig. 3a–d). Using μPET/μCT, we saw similar results with ^18^F-NaF binding only to the surface of macrocalcific deposits (Fig. 3e,f), matching the limits of penetration demonstrated with autoradiography.

One of the main aims of this work was to explain the previously observed differences between the PET and CT signals in the clinical setting. We chose to investigate this using preclinical μPET/μCT because of the higher spatial resolution that it offers (μPET, 900 μm; μCT, 30 μm). We hypothesized that the use of higher-resolution μPET/μCT would allow detection of microcalcifications. While microcalcifications identified on histology (Fig. 3g) were not detectable by μCT (Fig. 3i), they were identified by an increased ^18^F-NaF μPET signal (Fig. 3j) that closely matched phosphor-imaging autoradiography (Fig. 3h). Finally, we further investigated ^18^F-NaF penetration by examining the intensity and density of the μPET and μCT signals (where calcification was defined as ≥1,000 Hounsfield units (HU)) along 5–8-mm linear transects of atherosclerotic tissue (Fig. 3k–o). Once again there were clear differences in the signal produced by these two imaging techniques with increased ^18^F-NaF activity on the surface of macroscopic calcium deposits that by contrast was not observed within their core.

### Comparing clinical *in vivo* PET/CT and *ex vivo* μPET/μCT

**Figure 4:**
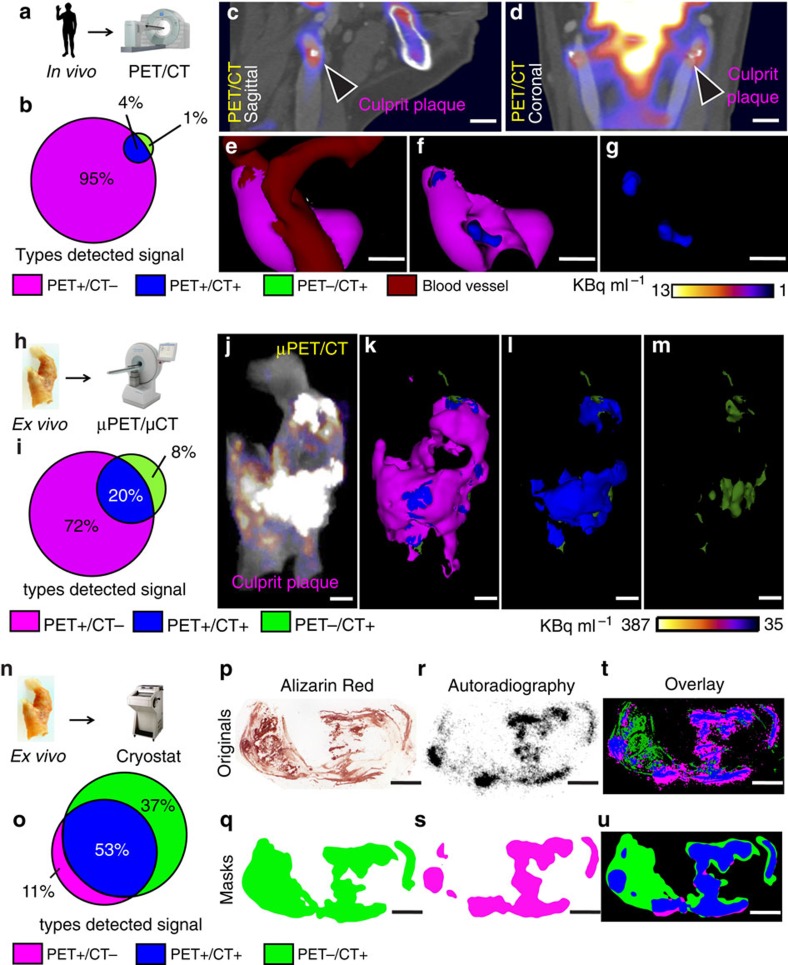
High-resolution imaging reveals specificity of ^18^F-NaF binding to vascular calcification. (**a**) Four symptomatic patients underwent clinical PET/CT imaging after injection of ^18^F-NaF and before carotid endarterectomy. (**b**) Comparison of PET and CT signals. Although clinical PET spatial resolution is much less than CT, PET detects larger area than CT, demonstrating higher sensitivity. PET+/CT−: magenta, 95%, PET+/CT+: blue, 4%, PET−/CT+: green, 1%. (**c**,**d**) Clinical PET/CT scan with the culprit atheroma (arrowheads). Scale bar, 11 mm (**c**) Sagittal view; (**d**) Coronal view. (**e**–**g**) Analysis of the carotid *in vivo*, where PET signal (magenta) was detected using observer-independent Otsu histogram-based thresholding. PET signal is co-localized with CT vascular calcification (blue). CT-detected soft tissue is dark red. Scale bar, 7 mm. (**h**) After endarterectomy, carotids were recovered, incubated in ^18^F-NaF and scanned in a μPET/μCT scanner. (**i**) Ratio of PET+/CT− area and CT+ signals is smaller than in **b**, due to the higher μPET resolution, which results in more precise signal detection. PET+/CT−: magenta, 72%, PET+/CT+: blue, 20%, PET−/CT+: green, 8%. (**j**–**m**) E*x vivo* images of the carotid from the same patient as in **c**–**g**. (**j**) μPET/μCT image without thresholding; (**k**–**m**) 3D thresholded μPET/μCT images. μPET signal (magenta), PET+/CT+ (blue), PET−/CT+ (green). (**n**) After μPET/μCT scan, carotids were incubated in ^18^F-NaF and sectioned on cryostat. (**o**) Ratio between PET+/CT− and CT+ regions indicates an even higher PET+/CT− resolution. PET+/CT−: magenta, 11%, PET+/CT+: blue, 53%, PET−/CT+: green, 37%. (**p**) Carotid from the same patient as in **c**–**g**, **j**–**m**. Alizarin Red detects both macro- and microcalcifications with the highest precision of all methods described here. (**q**) Alizarin Red image after Gaussian filter and Li thresholding, to match resolution to autoradiography image. (**r**) Autoradiography shows highly specific binding to all microcalcifications, but is not able to penetrate the deeper levels of macrocalcifications. (**s**) Autoradiography image after Gaussian filter and Li thresholding, to match resolution to the filtered Alizarin Red image. (**t**) Overlay of unfiltered histology and autoradiography images showing a high PET+/CT− signal, due to mismatch in resolution. (**u**) Overlay of filtered histology and autoradiography images shows matching signal resolution. Scale bar=3 mm. 3D movies in Supplementary Data.

We applied a novel approach to compare the *in vivo* and *ex vivo* imaging of carotid atherosclerotic plaque using co-registered clinical PET with CT, μPET with μCT and autoradiography with histology. In particular, we focused on the mismatch between the ^18^F-NaF PET signal and CT identifiable calcification (Fig. 4).

We recruited four patients who were scanned with ^18^F-NaF PET/CT before carotid endarterectomy (Fig. 4a). After surgery, we incubated the endarterectomy specimens with ^18^F-NaF and imaged them with the μPET/μCT scanner (Fig. 4h). Finally, we again incubated the carotid endarterectomy specimens with ^18^F-NaF and cryosectioned them to obtain autoradiography images and light microscope histology to detect areas of calcium (Fig. 4n). On the clinical PET/CT and μPET/μCT scans, we confirmed differences in the distribution of calcium on CT and ^18^F-NaF uptake on PET (Fig. 4c–g, for Fig. 4e–g corresponding three-dimensional (3D) Supplementary Movies 1,2,3). Regions of ^18^F-NaF uptake were most commonly observed in the absence of calcium on CT, representing areas of microcalcification (PET+/CT−; Fig. 4b,i). In addition, there were also large CT-detected areas of macrocalcification that did not demonstrate concomitant ^18^F-NaF uptake (PET−/CT+; Fig. 4b,g (for Fig. 4g, corresponding 3D Supplementary Movie 3)). These are likely to represent regions where nascent microcalcification is mild or absent.

The findings on μPET/μCT were similar to the clinical scans. Indeed the distribution of calcium detected with μCT closely matched that observed on the clinical CT although additional smaller areas of macrocalcification were also identified and resolved (Fig. 4j–m, corresponding 3D Supplementary Movies 4,5,6,7). The distribution of the ^18^F-NaF signal was also similar although again it was more refined on the μPET compared with the clinical PET systems (Fig. 4j–l, corresponding 3D Supplementary Movies 4,5,6). Finally, histology and ^18^F-NaF autoradiography provided the most sensitive method for the detection of tissue calcification (Fig. 4o–u). Histological staining with Alizarin Red confirmed that both μCT and clinical CT only detect regions of macrocalcification with extensive regions of microcalcification left unresolved by these techniques (Fig. 4p–u). By contrast, the ^18^F-NaF signal detected by autoradiography was observed in a very similar distribution to that observed by the μPET (albeit providing better spatial resolution), with both able to resolve regions of microcalcification (Fig. 4r–u). For the number of patients used in the individual experiments, see Supplementary Table 5.

## Discussion

For the first time, we demonstrate the specificity and sensitivity of ^18^F-NaF adsorption to calcium deposits in human atherosclerotic vascular tissue and demonstrate that it can non-invasively detect areas of microcalcification indicative of nascent calcification and active unstable atherosclerosis. This unique and characteristic signal has implications and ramifications for the non-invasive clinical imaging of human atherosclerosis—a condition that represents the leading cause of death worldwide.

In bone, the binding of fluoride to areas of calcification is mediated through a chemical reaction with hydroxyapatite, a crystalline structure that is also the main component of vascular mineralization^22^. Using electron microprobe analysis, we have shown for the first time that fluoride also closely co-localizes to pathological mineralization within vascular tissue. The precise target for NaF binding in living vasculature has been uncertain, as fluoride ions were not expected to have the selectivity as seen between ligands and their cognate receptors, and NaF could target molecules other than hydroxyapatite. Here we demonstrate that ^18^F-NaF co-localizes to areas of nascent calcification and potentially provides a novel non-invasive biomarker of this high-risk pathology. Moreover, we observed that there is preferential adsorbing of fluoride to microcalcification, a clinically more significant manifestation of vascular mineralization. The extent of fluoride adsorption depends on the surface area of the mineral^24^,^25^ and would be consistent with the proposal that the complex convex surface structure of microcalcifications allows more binding of fluoride than the relatively flat and smaller surface area of macrocalcification.

The association and dissociation of ^18^F-NaF to carotid endarterectomy specimens showed an exponential rise and decay of signal, with excellent contrast between activity in vascular tissue and the blood pool observed as early as 60 min following injection. Studies in artificial chemical models have shown that fluoride binding to hydroxyapatite also occurs in an exponential binding reaction^24^,^25^ and can be explained both by physico- and chemisorption^24^. This also appears as the most likely mechanism in human carotids but it is important to emphasize that not all calcification observed can be generalized to consist of hydroxyapatite. Previous clinical radiology reports have described an initial fluoride-bound water shell entrapment and a slow ion exchange^22^; however, no mechanistic studies to prove these assumptions have been published, at least to our knowledge. Therefore, we propose that our observations match the findings in artificial systems and the fluoride adsorption to hydroxyapatite in whole-carotid sections can be explained by physico- and chemisorption and the dissociation is a result of a washout, similar to what has been described in artificial systems^24^.

The mechanism of early micronodular intimal atherosclerotic calcification is not yet clearly understood although its genesis has been linked to, amongst others factors, inflammatory macrophages, inflammatory lipids, apoptosis, neovascularization and phenotypically altered vascular smooth muscle cells^3^. We have demonstrated that ^18^F-NaF binds specifically and sensitively to the final common pathway of precipitated micro nodules of pathological calcification (whether through active or passive mechanisms) and not directly to any of these putative earlier upstream triggers. This is in contrast to ^18^F-fluorodexoyglucose, which although linked to activated macrophages in the vasculature, may lack sensitivity and specificity for identifying inflammation specific activation^26^ and does not have the highly favourable pharmacokinetics of ^18^F-NaF^26^,^27^.

**Figure 5:**
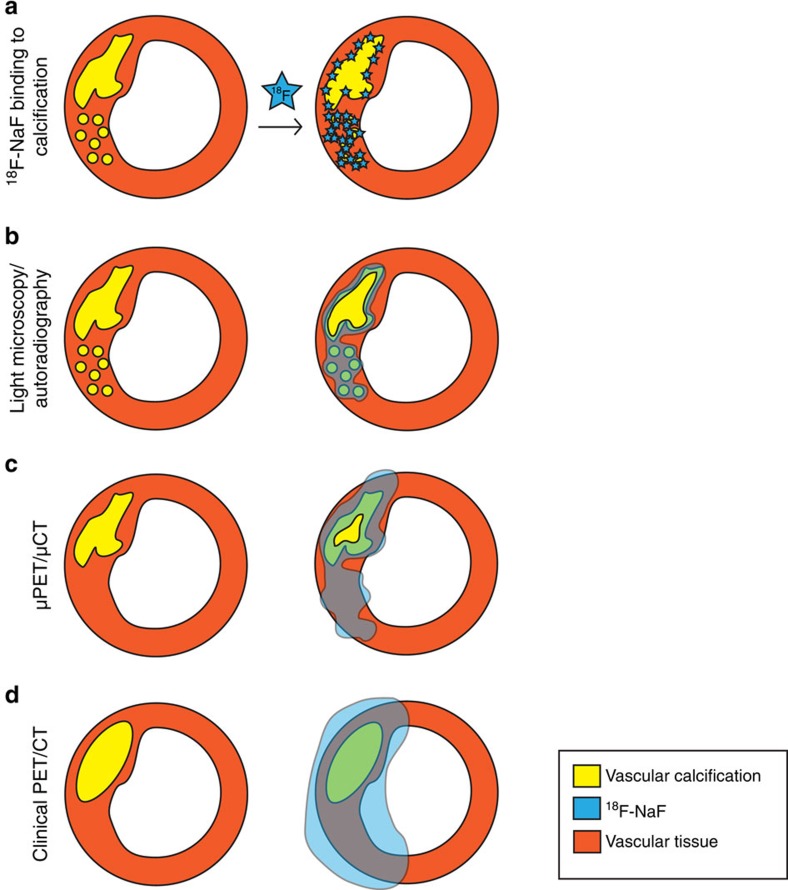
Our model of ^18^F-NaF binding to the vascular calcifications. (**a**) ^18^F-NaF highly specifically binds to both micro- and macrocalcifications and the signal strength depends on the available surface area of these calcifications. (**b**) Schematic of histology/autoradiography vascular calcification detection. Alizarin Red histology results in the most definitive delineation of calcification, with the detection limit into the nanometre range. Phosphor screen autoradiography also has a much higher resolution compared with PET and μPET, resulting in accurate detection of calcifications. (**c**) Schematic of preclinical μPET/μCT vascular calcification detection. μCT detects macrocalcifications and their finer architecture. Proportionally less signal is detected on μCT than using histology, yet more than the clinical CT. μPET very precisely detects both macro and microcalcifications. In addition, if the macrocalcifications exceed μPET resolution, it is possible to observe ^18^F-NaF binding to the outer surface of macrocalcifications. There are less PET−/CT+ regions observed than using autoradiography but more than clinical PET/CT. (**d**) Schematic of clinical PET/CT vascular calcification detection. Here CT is able to detect gross macrocalcifications and PET detects both CT+ and CT− calcifications. However, the signal is diffuse, resulting in much larger and therefore less precise PET+ detections, as compared with the *ex vivo* imaging modalities described here.

The main question we sought to answer was why does the ^18^F-NaF clinical PET/CT imaging result in a signal mismatch between the two modalities? Specifically, why do some areas of macrocalcification show no uptake, while other areas without apparent calcification show high ^18^F-NaF uptake? To address this, we used carotid endarterectomy sections and compared *ex vivo* and *in vivo* imaging approaches. Since vascular calcification consists predominantly of mineral and only a small amount of soft tissue, the architecture of the calcification in the *in vivo* and *ex vivo* environments was very similar. Using autoradiography, we were able to show that if the *ex vivo* carotid was first incubated in ^18^F-NaF and then cryosectioned, ^18^F-NaF only bound to the outer surface of the macrocalcifications. In contrast, microcalcifications had a greater surface area and no barriers to penetration of the tissues resulting in high levels of ^18^F-NaF adsorption. This suggests that ^18^F-NaF cannot penetrate into the deeper layers of macrocalcification. Since macrocalcifications have a large volume but small surface area, the detected radioactivity signal is proportionally smaller than microcalcifications with a small volume and large surface area. Microcalcifications are also dispersed in large numbers per given area of the tissue, again resulting in a disproportionally high signal compared with macrocalcifications. Furthermore, when sectioned carotid endarterectomy specimens were directly exposed to ^18^F-NaF, uptake was this time observed also in the centre of macroscopic deposits that had now been made accessible to the tracer. We therefore conclude that the adsorption of ^18^F-NaF to areas of calcification in carotid endarterectomy tissue is a measure of the available surface area accessible to the isotope. This is consistent with findings in the bone and explains the observed greater adsorption of ^18^F-NaF to microcalcifications (Fig. 5a,b).

μPET/μCT scans of *ex vivo* carotids and their comparison with autoradiography revealed that μPET closely matches autoradiography. On the other hand, μCT was only able to detect macrocalcifications (Fig. 5c) and underestimated the extent of vascular microcalcification identified by Alizarin Red histology. Back-correlation of μPET/μCT *ex vivo* scans to clinical *in vivo* PET/CT scans showed similar results. Using clinical CT, it was possible to detect the larger macrocalcifications, and their shape closely matched the data acquired by μCT. In contrast, the ^18^F-NaF uptake detected by the clinical and μPET co-localized with both underlying calcium deposits (PET+/CT+) and regions with no apparent calcification (PET+/CT−) as assessed on CT (Fig. 5d). For the latter situation, our histological studies showed that the PET signal was in fact binding to microcalcifications below the detection limit of clinical CT and μCT. We therefore conclude that areas of ^18^F-NaF uptake are reporting underlying microcalcifications, which are undetectable by CT. Conversely, large CT-detected macrocalcifications that do not co-localize with ^18^F-NaF PET uptake (PET−/CT+) might be considered to represent dormant areas where on-going mineralization, as evidenced by the presence of microcalcification, has ceased and atherosclerotic disease is quiescent. Indeed, more heavily calcified plaques have been reported to be particularly prevalent in more stable disease and are much less vulnerable to rupture^28^,^29^.

In summary, using electron microprobe analysis, we have demonstrated for the first time that fluoride directly adsorbs to calcified areas in mineralized vascular tissue. We have shown that binding is highly specific since ^18^F-NaF radioactivity is confined solely to calcification, with no localization to other soft tissues. Furthermore, we observed that ^18^F-NaF signal is highly dependent on the surface area of the calcification, being able to adsorb only to the outer layer of macrocalcifications without deeper penetration. We replicated these findings on three levels—autoradiography, light microscopy, *in vivo* clinical PET/CT and *ex vivo* μPET/μCT. These characteristic position ^18^F-NaF as a highly specific ligand for the detection of pathologically high-risk microcalcification and early unstable atherosclerotic disease. ^18^F-NaF is an economical PET ligand that is easy to manufacture. If used clinically, ^18^F-NaF uptake has the potential to identify microcalcification within high-risk plaques as well as determine the locations of plaque rupture. In addition, as a marker of nascent calcification, it could test the efficacy of pharmacological therapies targeting atherosclerosis or calcification in both the clinical and preclinical settings. Microcalcifications are also linked to other disease processes, such as breast cancer, prostate cancer and stroke. The main aim of this study was to understand the molecular mechanisms of ^18^F-NaF uptake and not to define patients at risk. With data validating its co-localization with calcification, the potential now exists to evaluate these processes. Whether or not ^18^F-NaF will be useful in stratifying risk of cardiovascular events remains to be demonstrated. Large-scale prospective investigations will be essential to determine the prognostic value for ^18^F-NaF role in risk stratification. Nevertheless, ^18^F-NaF PET/CT holds promise and may have wide-ranging applications and become a valuable clinical tool to study active calcification across many disease areas and disciplines.

## Methods

All chemicals were purchased form Sigma-Aldrich, Dorset, UK, unless stated otherwise.

### Human tissues

The atherosclerotic intimal layers of human carotid arteries (further in text: carotids) were obtained with ethical approval and informed consent after endarterectomy surgeries (National Health Service Local Research Ethics Committee approval in Cambridge: 97/084, National Health Service West of Scotland Research Ethics Committee approval in Edinburgh: 12/WS/0227). The mean patient age was 71±2 years and both male and female patient carotids were used. The tissue was collected immediately after the surgery and was fresh frozen in −80 °C. It was kept frozen until the start of the experimental procedures. In clinical imaging, the concentration of the injected ^18^F-NaF in plasma was calculated as approximately 1 × 10^−11^ M. Initial experiments were performed both on fresh carotids and carotids that had undergone a freeze-thaw cycle and no differences in ^18^F binding were observed in terms of radioactivity penetration into macrocalcifications.

### Electron microscopy

Frozen carotid tissue was placed in cryostat for 1 h to equilibrate to −20 °C temperature. It was then immersed in Optimal Cutting Temperature compound (OCT) Embedding Matrix (CellPath, Powys, UK) and 30-μm thick, serial sections were cut on a Bright (Huntingdon, UK) cryostat. Sections were placed on custom-made Melinex discs (diameter: 12 mm) and allowed to dry. Discs were then frozen in −80 °C temperature until the day of experiment. During the experiment, tissue sections were allowed to thaw and then incubated with 120 μl 0.01 M non-radioactive NaF for 2 h at room temperature. After incubation, sections were dipped in distilled H_2_O three times and allowed to dry. The discs were stuck to 12.5 mm Cambridge style scanning electron microscope stubs and coated with carbon in an Edwards Auto 306 evaporative carbon coater. They were imaged in an FEI XL30 FEGSEM operated at 20 kV with secondary and backscattered electron detectors. Calcification was detected predominantly in the regions of intimal thickening and was designated micro- (spherical or near spherical deposits <50 μm in diameter) or macrocalcification (where large regions between 50 μm and several mm in size were calcified). Both types of calcification were analysed by energy-dispersive X-ray microanalysis in 10 arteries. Spectra were collected using an Oxford Instruments SiLi atmospheric thin window detector running INCA software (ETAS Group, Stuttgart, Germany). Between 10 and 57 spectra were collected from both types of calcification (where present) for 100 s live time. As a control to assess nonspecific binding of F, spectra were also collected from regions of the arterial media where no backscattered electron signal for hydroxyapatite was present. The spectra were deconvolved in the INCA software and to estimate F/Ca only Ca and F spectra were input into the analysis. The quantitative data were expressed as atomic %. F/Ca was estimated by dividing the atomic % of F by the atomic % of Ca. Data are presented as means±the s.e.m. and F/Ca between regions of micro- and macrocalcification was compared using an ANOVA and Tukey Kramer *post hoc* test.

Additional electron microscopy analysis was performed with commercial hydroxyapatite standards. Hydroxyapatite (2 mg) was incubated with 1 ml of 1% NaF on a rotator for 8 h or 4 days. The standards were then rinsed 10 times for 20 min in distilled water and air dried onto coverslips. They were mounted onto 12.5 mm Cambridge scanning electron microscope stubs, carbon coated and analysed in the same way as the cryostat sections. Control samples of hydroxyapatite standards were treated similarly as described above but the NaF incubation step was omitted. The hydroxyapatite analysed in the cryostat section is at least partially embedded/encapsulated in the tissue of the arteries and sections were coated in the same way with carbon to make them electrically conductive. As the carbon was added it was excluded from the analysis. Concentrations of O, P and Ca in the control hydroxyapatite are expressed in atomic per cent.

In the NaF-treated hydroxyapatite, no signal for Na was seen after extensive washes, and the concentrations of O, F, P and Ca were expressed as atomic per cent. Despite extensive washing, a clear signal for F was retained, indicating binding to the hydroxyapatite. In these control samples the interaction volume of the electron beam is expected to remain within hydroxyapatite alone whereas in the tissue sections a proportion of the interaction volume will also generate X-rays from the surrounding arterial tissue. Therefore the ratio of F/Ca was used to measure differences in F concentration between areas of macro- and micro- calcification.

### ^18^F-NaF binding to cryostat cut carotid artery sections

For all experiments ^18^F-NaF was synthesized by the Wolfson Brain Imaging Centre (WBIC) (Cambridge, UK). The typical activity was approximately 285 MBq.

Frozen whole carotids were placed in the cryostat for 1 h to equilibrate to −20 °C temperature. Tissue was then immersed in the OCT Embedding Matrix (CellPath, Powys, UK) and 20 μm thick, serial sections were cut on a Bright (Huntingdon, UK) cryostat. Sections were allowed to dry in room temperature and stored at −80 °C temperature until the start of the experiment.

The concentration of ^18^F-NaF was detected using F^−^ electrode (Thermo Scientific, Loughborough, UK) and was then diluted to 1 × 10^−11^ M. For nonspecific binding detection, 20 μl of 0.1 M unlabelled NaF solution (Orion Ionplus F^−^ Standard, Thermo Scientific, Loughborough, UK) were added to an aliquot of the radioactive 1 × 10^−11^ M solution. Frozen tissue sections were allowed to thaw and washed with PBS for 10 min in room temperature. Adjacent sections were incubated either with ^18^F-NaF or ^18^F-NaF plus unlabelled NaF solutions for 1 h at room temperature. They were then washed in PBS 3 × 5 min and dipped in distilled H_2_O. After drying, tissues were placed on a charged phosphor screen (Perkin Elmer, Waltham, Massachusetts) and left overnight. The following day screens were read using PerkinElmer's Cyclone Plus Phosphor Imager (Waltham, Massachusetts) and data were analysed with OptiQuant software (Packard Instrument, Meriden, Connecticut). Areas of micro- and macrocalcification were identified visually based on the size of nodules of <50 and ≥50 μm to several mm, respectively, and manual measurements made.

For concentration–response curve, the same method was used, except stock ^18^F-NaF solution was split into serial dilutions from 10^−12^ to 10^−7^ M. After washing in PBS and dipping in distilled H_2_O, tissues were wiped off the slides with filter paper, which was put into radioimmunoassay tubes and measured in the gamma counter (Packard Cobra II E5003, GMI, Ramsey, Minnesota).

### ^18^F-NaF binding in whole-carotid arteries

Whole-carotid arteries were thawed in 5 ml PBS for 1 h, and then placed in 5 ml of 10^−11^ M ^18^F-NaF solution for 1 h at room temperature. They were then washed in PBS 3 × 2 min, followed by a dip in distilled H_2_O. Carotids were transferred to cryostat, immersed in OCT and allowed to freeze. Tissue was then cut into 20-μm serial sections, which were allowed to dry on microscopy slides. After drying, tissues were placed on a charged phosphor screen and left overnight.

For whole-carotid association and dissociation binding curves, the same method was used, except during association binding experiments, carotids were taken out from the 5 ml of 10^−11^ M ^18^F-NaF tube at set time points—0, 5, 15, 30, 60 min, washed and measured in the gamma counter. For dissociation binding experiment, after the incubation in 5 ml of 10^−11^ M ^18^F-NaF for 1 h, tissues were continuously washed in PBS for set amounts of time—0, 5, 10, 15, 30, 45, 60, 90, 120 min. Instead of cryostat cutting, arteries were placed in radioimmunoassay tubes and measured in the gamma counter.

### Tissue staining

Tissue sections were cut on cryostat as described in section ‘^18^F-NaF binding to cryostat cut carotid artery sections‘.

### Alizarin red staining

Alizarin Red staining (Alfa Aesar, Heysham, UK) was used for calcification detection in carotid arteries. In brief, cryosections were left to dry overnight. They were then fixed in acetone for 10 min at 4 °C and washed in PBS 2 × 5 min at room temperature. After washing, 300 μl Alizarin Red were applied to each section for 1 min. Tissue was then put in acetone for 1 min, followed by a wash in 50:50 acetone:xylene for 1 min and then left in xylene for at least 1 h.

### Immunohistochemistry

Immunohistochemistry was used to detect multiple inflammatory tissue markers in cryosections. Tissue was left to dry overnight and the following day it was fixed in acetone at 4 °C for 10 min and washed in PBS 2 × 5 min at room temperature. Sections were blocked in 10% goat serum for 1 h and H_2_O_2_ for 5 min. Each blocking step was followed by washing in PBS for 2 × 5 min. Carotids were then incubated in primary antibody for 30 min at room temperature. Antibodies included CD68 (1:500, clone PG-M1), CD31 (1:20, clone JC70A) and Smooth Muscle Actin (1:500, clone 1A4) (all from Dako UK, Ely, UK). Labelled polymer-HRP anti-rabbit application and visualization with 3,3′-diaminobenzidine (DAB) was done using EnVision+ System-HRP (DAB) kit (Dako UK, Ely, UK), following manufacturers specifications. Counterstain of 150 μl haemotoxylin was applied to each section and incubated for 1 min. Slide was then dipped six times in destain solution (1% HCl, 50% methanol, 49% distilled H_2_O) and washed in tap water for 2 min. Afterwards tissue was quickly dehydrated in 1 min intervals through graded alcohols (30 to 70% to 100% to absolute alcohol 1 to absolute alcohol 2) and cleared in xylene for at least 1 h.

### Section mounting

After staining, all tissues were mounted using DePeX mounting medium Gurr (VWR, Lutterworth, UK) and glass coverslips (Menzel-Gläser, Braunschweig, Germany). After hardening of the mounting solution, tissues were imaged using Wild Heerbrugg M3Z microscope (Leica, Heerbrugg, Switzerland).

### μPET/μCT imaging of whole-carotid arteries

Whole-carotid arteries were removed from −80 °C freezer and thawed in 5 ml PBS at room temperature for 1 h. After thawing, tissues were placed in 1 × 10^−9^ M ^18^F-NaF solution and incubated for 1 h. Tissues were then washed in PBS 3 × 2 min and dipped in distilled water. They were then placed in sealed plastic tubes. Tissue radioactivity was read using dose calibrator and they were then placed into the μPET/μCT scanner. Images were acquired over 30 min in list mode using a NanoScan PET/CT (Mediso, Budapest, Hungary). Data were reconstructed using a 3D ordered-subset expectation-maximization (OSEM) algorithm with eight iterations and six subsets using corrections for decay, dead time, random events, attenuation and scatter, the resulting image was isotropic with 300 μm voxels. Two sets of μCT were performed immediately after μPET acquisition, using high and low zoom (67.1 mm and 219 mm between object and source, respectively). Images were obtained by helical acquisition using a voltage, current and exposure time of 65 kVp, 123 μA and 1,100 ms. Images were reconstructed using a modified cone beam filtered-back projection method using a Butterworth filter producing a 22-μm and a 213-μm isotropic data set. μPET and μCT data were co-registered using previously established default shifts.

### 2D image processing and analysis

Two-dimensional image analysis was performed using open-source software ImageJ/Fiji (http://fiji.sc) (NIH, Bethesda, Maryland). Correlation between histology sections and autoradiography images was achieved by using thresholded binary images of the different signals. In addition, these thresholded images were filtered with Gaussian blur (*σ*=20) and thresholded again, to create segmentation masks that overcome the issue of inherent resolution differences between histology and autoradiography signal detection processes^30^.

Alizarin Red-stained calcification detection was performed using histogram-based Otsu automated thresholding method^31^ and after Gaussian filter, it was processed using Li thresholding^32^.

DAB-stained immunohistology sections were analysed using a colorimetric thresholding method. While calcification stains provided excellent signal-intensity to background ratio, this was not the case for immunohistochemistry. Therefore, a colour combination that only detected the brown DAB stain was set as the manual threshold value, irrespective of intensity. After Gaussian blurring, signal was detected using Li auto-thresholding method.

Autoradiography images were resized to match the size of the histology images. Then they were thresholded using Otsu method, Gaussian blurred and thresholded using Li method.

All two-dimensional histology images were subjected to background subtraction and histogram equalization.

Correlation between the various stained sections was accomplished using multiple ImageJ/Fiji plug-ins. The position of specimens between individual images was co-registred using TurboReg^33^ plug-in. Although tissues had been exposed to acetone and alcohol fixation protocols, we did not observe prominent contraction or shrinkage that would affect our data analysis. Images were then thresholded and turned into binary data as described above. Pearson's correlation coefficient was calculated using JACoP^34^ plug-in. Overlapping images of histological and autoradiography signals were acquired using Co-localization Finder plug-in. The overlapped images were processed further to determine PET+/CT−, PET+/CT+ and PET−/CT+ regions in individual sections. Each colour corresponding to one of the three detection modalities was separated out and quantified using Analyze Particles command which counts the number of pixels in the binary segmented mask.

### 3D image processing and analysis

3D images after data reconstruction were analysed using VivoQuant ver 1.23, (InviCRO, Boston, Massachusetts) software^35^. For μPET/μCT initially the files were cropped to reduce file size and remove experimental artifacts, such as the scanning panel and tube in which the carotid was placed. Once an image containing only the signals associated with the carotid was achieved, the colour intensities of both modalities were manually adjusted to match histology/autoradiography signals as close as possible. After that, Distance/Annotation function was selected and 5–8-mm linear transects were made across slides in the transversal plane of carotids. The raw data were then downloaded and graphs showing μCT densities and μPET units along the transect lines were later reconstructed with Matlab (Mathworks, Natick, Massachusetts) software. To quantify PET+/CT−, PET+/CT+ and PET−/CT+ regions on the 3D level, 3D ROI Tool function was selected. First, μPET data were thresholded using Otsu method, to delineate the extent of PET+ signal. On the μCT plane we defined densities over 1000 Hounsfield Units (HU) as calcifications. To detect PET+/CT+ signal, we selected PET+ delineated 3D area and used Global thresholding for ≥1,000 HU of the μCT data in this area. To detect PET−/CT+ areas, we selected all remaining μCT data and applied the ≥1,000 HU Global threshold. The raw data containing information about the different modality regions was downloaded and analysed with Microsoft Office Excel (Microsoft, Redmond, Washington).

Clinical PET/CT data were analysed by initially manually co-registering the PET, CT and CT angiogram using bones as anatomical landmarks of the three different modalities. Following this the images were zoomed in to the culprit plaque and was cropped around this area. The division into PET+/CT−, PET+/CT+ and PET−/CT+ regions was similar to as described for the μPET/μCT data. The only exception was that CT and CT angiogram CT+ regions were also detected using Otsu thresholding, to account for the inconsistencies of the amount of tracer injected and imaging time after ^18^F-NaF injection.

### Clinical PET/CT acquisition and dynamic analysis

Recruitment and scanning for the *in vivo* aspect of this study took place following approval from the local research ethics committee (National Health Service West of Scotland Research Ethics Committee approval in Edinburgh: 12/WS/0227) and in accordance with the Declaration of Helsinki. Patients with symptomatic carotid artery stenosis (scheduled to undergo carotid endarterectomy) ≥50% by NASCET criteria^36^ were recruited from stroke and vascular surgery clinics at the Royal Infirmary of Edinburgh between January 2013 and April 2014. Exclusion criteria included a modified Rankin score of ≥3, insulin-dependent diabetes mellitus, women of child-bearing age not receiving contraception, severe chronic kidney disease (epidermal growth factor receptor <30 ml min^−1^ per 1.73 m^2^), known iodine-based contrast allergy, prior ipsilateral carotid intervention, prior neck irradiation and inability to provide informed consent.

After giving their consent, patients underwent baseline clinical assessment before undergoing dynamic or ^18^F-NaF PET/CT and CT carotid angiography with the use of a hybrid scanner (Biograph mCT, Siemens Medical Systems, Erlangen, Germany).

For static ^18^F-NaF PET/CT, a target dose of 250 MBq of ^18^F-NaF was administered intravenously. Scanning took place 60 min after injection. An attenuation-correction CT scan (non-enhanced, low dose 120 kV, 50 mAs) was then performed followed by PET imaging covering two bed positions with the first upper bed centred over the carotid bifurcation in 3D mode for 15 min per bed. Analysis was undertaken as described above.

In five patients, ^18^F-NaF PET imaging was undertaken as a dynamic acquisition with the PET scanner in list mode. Three of these subjects underwent arterial blood sampling to directly assay the plasma arterial input function for model creation and to assess ^18^F-NaF pharmacokinetics. An image-derived input function was utilized for modelling in the two patients that did not undergo arterial cannulation and sampling. The image-derived input function was generated by drawing regions of interest in the proximal carotid artery lumen. After the fitting of a soft neck collar to minimize movement, subjects were placed in the PET/CT scanner with intravenous and radial arterial catheters sited. An attenuation-correction CT scan (non-enhanced, low dose 120 kV, 50 mAs) was performed followed by PET imaging with the single bed centred over the carotid bifurcation. A target dose of 250 MBq of ^18^F-NaF was then administered intravenously at the same time as scanning and blood sampling were initiated. Scanning took place for 75 min. Real-time whole-blood radioactivity was assayed using a calibrated Allogg ABSS V3 system (Allogg Technology, Mariefred, Sweden). In brief, whole arterial blood was sampled through a narrow bore polytetrafluoroethylene tube using a roller pump at a rate of 5 ml min^−1^ before being drawn through a shielded scintillation-based detector. Counts were recorded using a data-logger. After 15 min, the pump was deactivated and 5 ml samples were drawn intermittently for the remaining 60 min. Decay corrected whole blood and plasma radioactivity was then assayed using a gamma counter. The fractional difference between whole blood and plasma radioactivity was then calculated to adjust the data for the first fifteen min from the Allogg device.

Following static and dynamic PET acquisitions, CT carotid angiograms were also undertaken (Care Dose 4D, 120 kV, 145 mA, rotation time 0.5 s, pitch 0.8).

Dynamic imaging data were reconstructed using the Siemens Ultra-HD algorithm (time of flight + True X) with corrections applied for attenuation, dead time, scatter and random coincidences. Imaging data were then parsed into the following time bins as per Hawkins and Frost^37^,^38^ (12 × 10 s, 4 × 30 s, 12 × 240 s) and analysed on a PMOD workstation (PMOD v3.408, PMOD Technologies Ltd., Zurich, Switzerland).

For analysis, the image data were first reviewed for evidence of ^18^F-NaF uptake, image quality and patient movement. The CT angiogram was scrutinized for plaque presence, location and characteristics. The PET data were then resliced as part of automated registration to the CT data. Volumes of interest were then generated by drawing regions of interest on sequential axial slices on the registered data sets. Volumes were drawn to separately incorporate internal carotid artery plaque (where present) and a vertebral body. Vertebral data were obtained as a comparator and also as a way of ensuring our data were commensurate with the work of others. Tissue activity curves were then generated and assessed.

Dynamic analysis of fluoride uptake in the bone has been well described. Since, our data and that of others shows that over the time course of a typical ^18^F-PET/CT scan fluoride dissociation from calcified tissue is undetectably slow, the Patlak technique^39^,^40^ may be used to gain an estimate for the net flux of fluoride (*K*_*i*_) into a region of interest. Patlak derived *K*_*i*_ is calculated as follows:


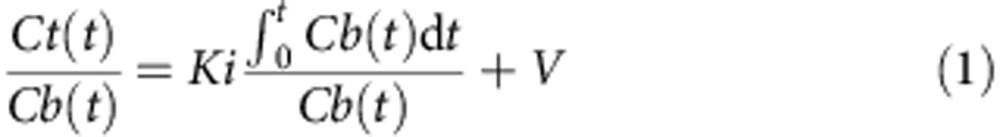


*C*_*t*_*(t)* is the activity concentration of ^18^F-NaF at time *t* within the volume of interest, *C*_*b*_*(t)* is the blood activity concentration of ^18^F-NaF at time *t* and *V* relates to the effective volume of distribution for ^18^F-NaF. The Patlak plot is obtained by plotting 
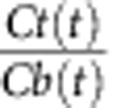
 against 
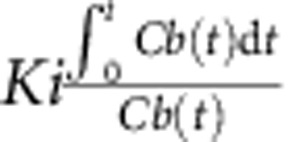
 and calculating the slope of the regression line as given in equation (1).

This method has been extensively described and compared with multi-compartmental modelling with both techniques showing good agreement. To ensure equilibrium between the plasma and compartments had been achieved, we only plotted the data from 10 min onwards as described by Frost^38^. Patlak plots were generated for all carotid plaques and vertebra (see Supplementary Fig. 2 for a representative example and Supplementary Table 1). We also plotted directly measured plasma activity curves for illustration of the pharmacokinetics of ^18^F-NaF (for a representative example, see Supplementary Fig. 3).

### Data analysis

Numerical data analysis was performed using Microsoft Office Excel (Microsoft, Redmond, Washington), Matlab (Mathworks, Natick, Massachusetts) and Prism (GraphPad, San Diego, California) softwares. Written text was produced using Microsoft Office Word (Microsoft, Redmond, Washington) and figures were made using Adobe Illustrator CS5 (Adobe Systems, San Jose, California).

## Additional information

**How to cite this article:** Irkle, A. *et al*. Identifying active vascular microcalcification by ^18^F-sodium fluoride positron emission tomography. *Nat. Commun.* 6:7495 doi: 10.1038/ncomms8495 (2015).

## Supplementary Material

Supplementary Figures and Tables Supplementary Figures 1-5 and Supplementary Tables 1-5

Supplementary Movie 1 3D image corresponding to Figure 4e, showing a human carotid artery in vivo, following injection with ^18^F-sodium fluoride and subsequently imaged using clinical PET/CT, where the amount of PET signal (magenta) was detected using observer-independent Otsu histogram-based thresholding

Supplementary Movie 2 3D image corresponding to Figure 4f, showing the same human carotid artery in vivo, as Movie 1 where PET signal is shown in magenta and CT vascular calcification in blue

Supplementary Movie 3 3D image corresponding to Figure 4g showing the same human carotid artery in vivo, as Movie 1 where CT signal is shown in blue

Supplementary Movie 4 3D image corresponding to Figure 4j showing a human carotid artery ex vivo, following incubation with 18F-sodium fluoride and subsequently imaged using μPET/CT, showing μPET/μCT image without thresholding

Supplementary Movie 5 3D image corresponding to Figure 4k showing the same human carotid artery ex vivo as Movie 4, showing 3D thresholded μPET/μCT segments

Supplementary Movie 6 3D image corresponding to Figure 4l of the same human carotid artery ex vivo as Movie 4, showing 3D thresholded μPET/μCT segments

Supplementary Movie 7 3D image corresponding to Figure 4m of the same human carotid artery as Movie 4, showing 3D thresholded μPET/μCT segments
