# Supplementary material for: Identifying active vascular microcalcification by 18F-sodium fluoride positron emission tomography
Source: Nat Commun. 2015 Jul 7;6:7495. doi: 10.1038/ncomms8495 (PMC4506997; doi:10.1038/ncomms8495)
Supplement: Supplementary Figures and Tables — Supplementary Figures 1-5 and Supplementary Tables 1-5 [file ncomms8495-s1.pdf]

**Supplementary Information**

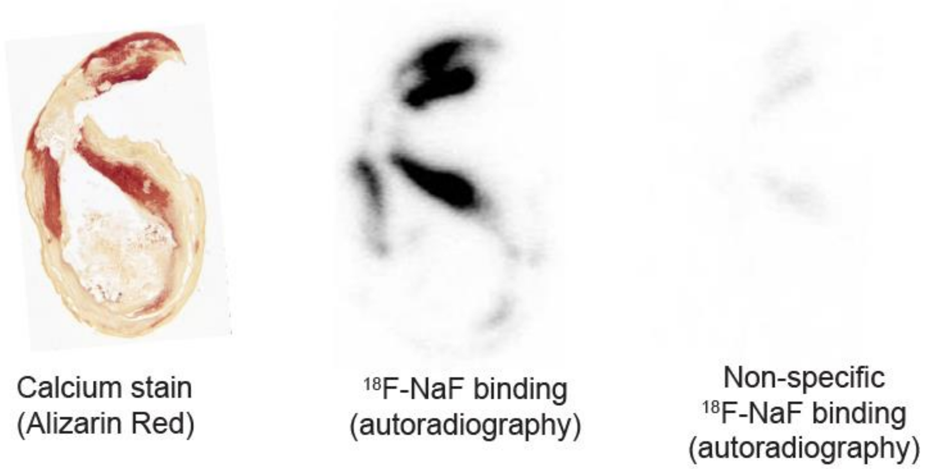


**Supplementary Figure 1** The non-specific binding of $^{18}$F-NaF is low.

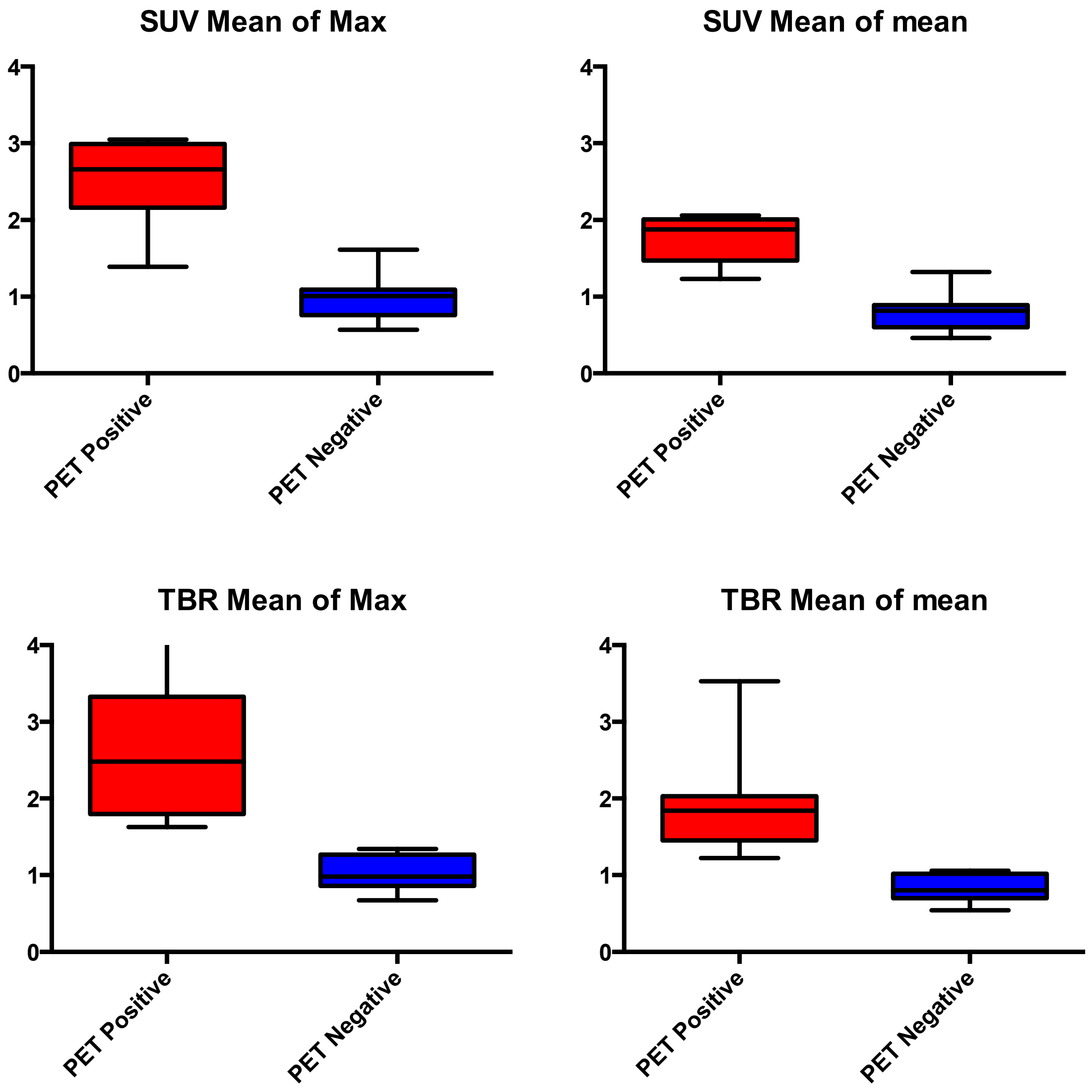


**Supplementary Figure 2** Quantification of $^{18}$F-NaF Uptake On Clinical Imaging. Box and whisker plots of Standardised Uptake Values and Target to Background Ratios for PET positive regions (red) and PET negative regions (blue) (Boxes show interquartile range, whiskers show range).

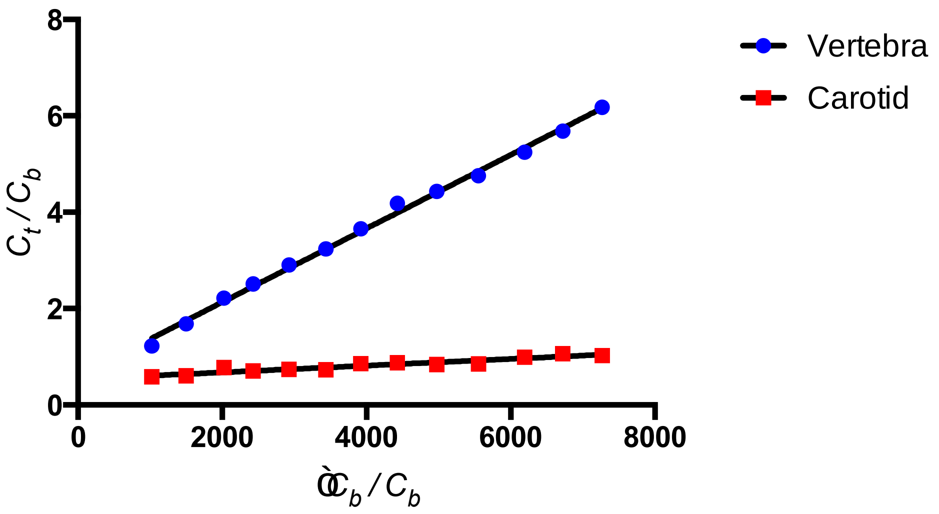


**Supplementary Figure 3** Dynamic PET Analysis. Representative Patlak plots from a single subject showing vertebral and carotid uptake where $C_b$ = plasma counts & $C_r$ = counts within the region of interest.

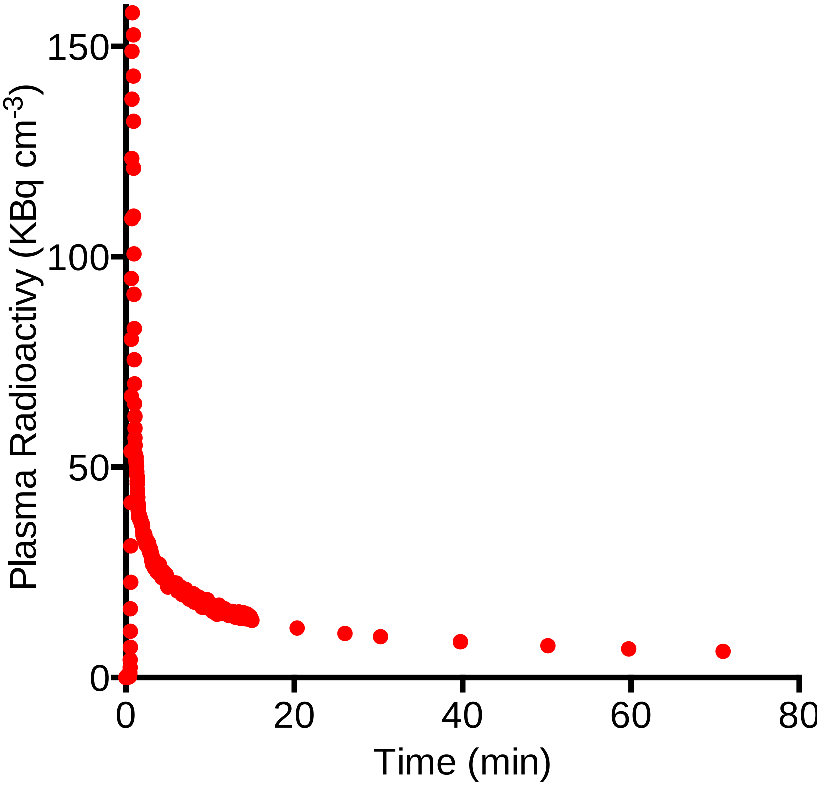


**Supplementary Figure 4** $^{18}$F-NaF Pharmacokinetics. A typical arterial plasma activity curve (from a single patient) showing a very rapid redistribution and clearance of $^{18}$F-NaF from the circulation.

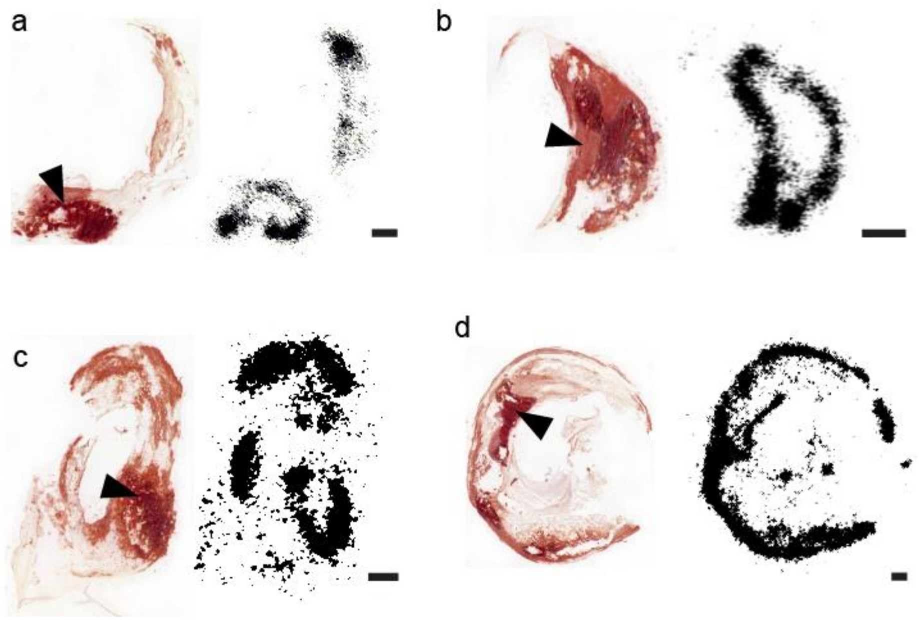


**Supplementary Figure 5** Additional representative examples of $^{18}F$-NaF inability to penetrate deeper layers of macro-calcifications (closed arrowheads). Scale bar = 100 μm.

| | O | P | Ca |
|---|---|---|---|
| Mean ± SD | 77.76 ± 7.21 | 9.26 ± 2.73 | 12.98 ± 4.74 |

Native Sigma hydroxyapatite. Element concentration in atomic % (n= 51).

| | O | P | Ca | F |
|---|---|---|---|---|
| Mean ± SD | 76.01 ± 2.58 | 5.30 ± 2.78 | 7.48 ± 0.79 | 11.21 ± 1.30 |
| F/Ca Mean ± SD | 0.70 ± 0.26 | | | |

Sigma hydroxyapatite after incubation with 1% NaF for 8 hours. Element concentration in atomic % (n= 51).

| | O | P | Ca | F |
|---|---|---|---|---|
| Mean ± SD | 73.44 ± 2.81 | 7.91 ± 2.87 | 7.37 ± 0.73 | 11.28 ± 1.20 |
| F/Ca Mean ± SD | 0.90 ± 0.24 | | | |

Sigma hydroxyapatite after incubation with 1% NaF for 4 days. Element concentration in atomic % (n= 51).

**Supplementary Table 1** EM analysis of hydroxyapatite standards to confirm co-localization of NaF.

| Subject | Age | Sex | Neurovascular Syndrome | Duplex NASCET Stenosis | Days between symptoms and PET scan | Static or Dynamic Scan | Used for Micro/Clinical Comparison |
|---|---|---|---|---|---|---|---|
| 1 | 88 | Male | Stroke | 65 | 10 | Static | Yes |
| 2 | 73 | Female | TIA | 80 | 13 | Static | Yes |
| 3 | 76 | Male | TIA | 85 | 26 | Dynamic | Yes |
| 4 | 59 | Male | TIA | 85 | 14 | Dynamic | Yes |
| 5 | 47 | Female | Stroke | 65 | 9 | Dynamic | No |
| 6 | 63 | Female | Asymptomatic | 60 | n/a | Dynamic | No |
| 7 | 76 | Male | Asymptomatic | 40 | n/a | Dynamic | No |

**Supplementary Table 2** Patient demographics and basic imaging data.

| Subject Number | PET Positive | | | | | PET Negative | | | |
|---|---|---|---|---|---|---|---|---|---|
| | SUV | | TBR | | | SUV | | TBR | |
| | Average Max | Average Mean | Average Max | Average Mean | | Average Max | Average Mean | Average Max | Average Mean |
| 1 | 2.66 | 1.47 | 3.33 | 1.84 | | 1.01 | 0.82 | 1.27 | 1.02 |
| 2 | 3.05 | 2.06 | 1.80 | 1.22 | | 1.61 | 1.32 | 0.95 | 0.78 |
| 3 | 2.75 | 1.94 | 2.48 | 1.75 | | 1.09 | 0.89 | 0.98 | 0.80 |
| 4 | 2.99 | 2.01 | 5.26 | 3.53 | | 0.76 | 0.60 | 1.34 | 1.06 |
| 5 | 1.39 | 1.23 | 1.63 | 1.45 | | 0.57 | 0.46 | 0.67 | 0.54 |
| 6 | 2.30 | 1.88 | 2.24 | 1.84 | | 0.88 | 0.71 | 0.86 | 0.70 |
| 7 | 2.16 | 1.70 | 2.57 | 2.03 | | 1.06 | 0.82 | 1.26 | 0.98 |

**Supplementary Table 3** Standardized Uptake Values and Target to Background Ratios for PET positive *versus* PET negative regions.

| | | Patlak | |
|---|---|---|---|
| | | **$K_i$ [ml/cm$^3$/min]** | **SE [%]** |
| **Subject 1** | Right Carotid | 0.0063 | 12.7 |
| | Left Carotid | 0.0060 | 10.3 |
| | Vertebra | 0.0486 | 3.0 |
| | | | |
| **Subject 2** | Right Carotid | 0.0042 | 10.5 |
| | Left Carotid | 0.0028 | 7.1 |
| | Vertebra | 0.0459 | 1.8 |
| | | | |
| **Subject 3†** | Right Carotid | 0.0005 | 24.0 |
| | Left Carotid | 0.0009 | 34.1 |
| | Vertebra | 0.0431 | 2.8 |
| | | | |
| **Subject 4*** | Left Carotid | 0.0045 | 21.6 |
| | Vertebra | 0.0694 | 1.9 |
| | | | |
| **Subject 5*** | Left Carotid | 0.0068 | 28.2 |
| | Vertebra | 0.0649 | 3.4 |

**Supplementary Table 4** Dynamic PET Analysis. $K_i$ values for individual carotid plaques and vertebra. SE is standard error. Subjects 4 and 5 had no plaque in their right internal carotid arteries. The vertebral values are in agreement with previously published data. † Subject 3 had very little carotid plaque uptake. * Subject 4 and 5 $K_i$ were estimated using image derived input functions.

| | *n*, individual patients |
|---|---|
| Electron microscopy | 10 |
| Concentration-response curve | 5 |
| Time-activity curve | 5 |
| Histology | 8 |
| Dynamic PET scan | 5 |
| Translatonal clinical PET/CT to µPET/CT to Autoradiography/histology | 4 |

**Supplementary Table 5** Summary of patients involved in individual experiments.
