# Supplementary figures and images for: Identifying active vascular microcalcification by 18F-sodium fluoride positron emission tomography
Source: Nat Commun. 2015 Jul 7;6:7495. doi: 10.1038/ncomms8495 (PMC4506997; doi:10.1038/ncomms8495)

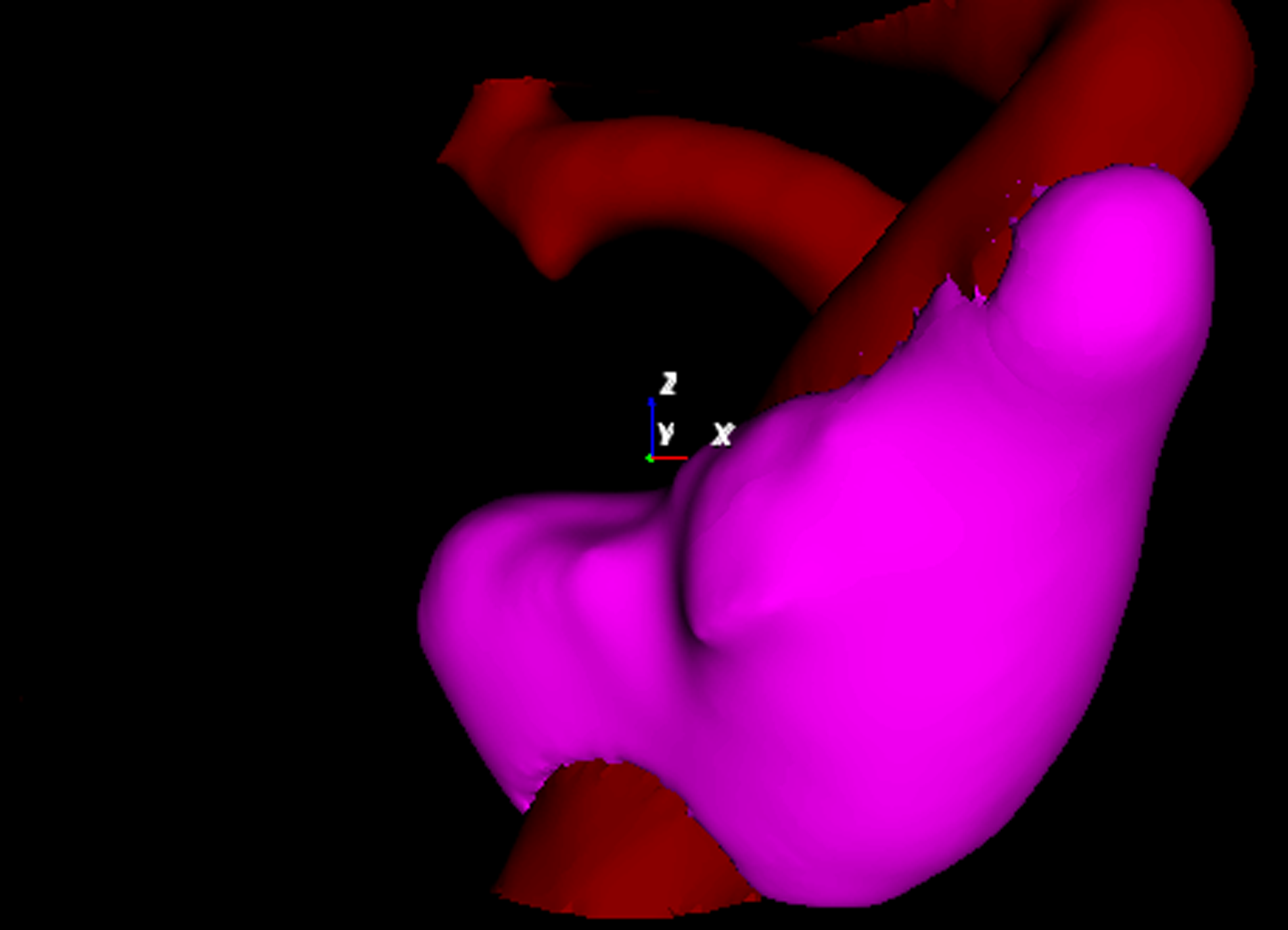

Supplement: Supplementary Movie 1 — 3D image corresponding to Figure 4e, showing a human carotid artery in vivo, following injection with 18F-sodium fluoride and subsequently imaged using clinical PET/CT, where the amount of PET signal (magenta) was detected using observer-independent Otsu histogram-based thresholding [file ncomms8495-s2.tif]

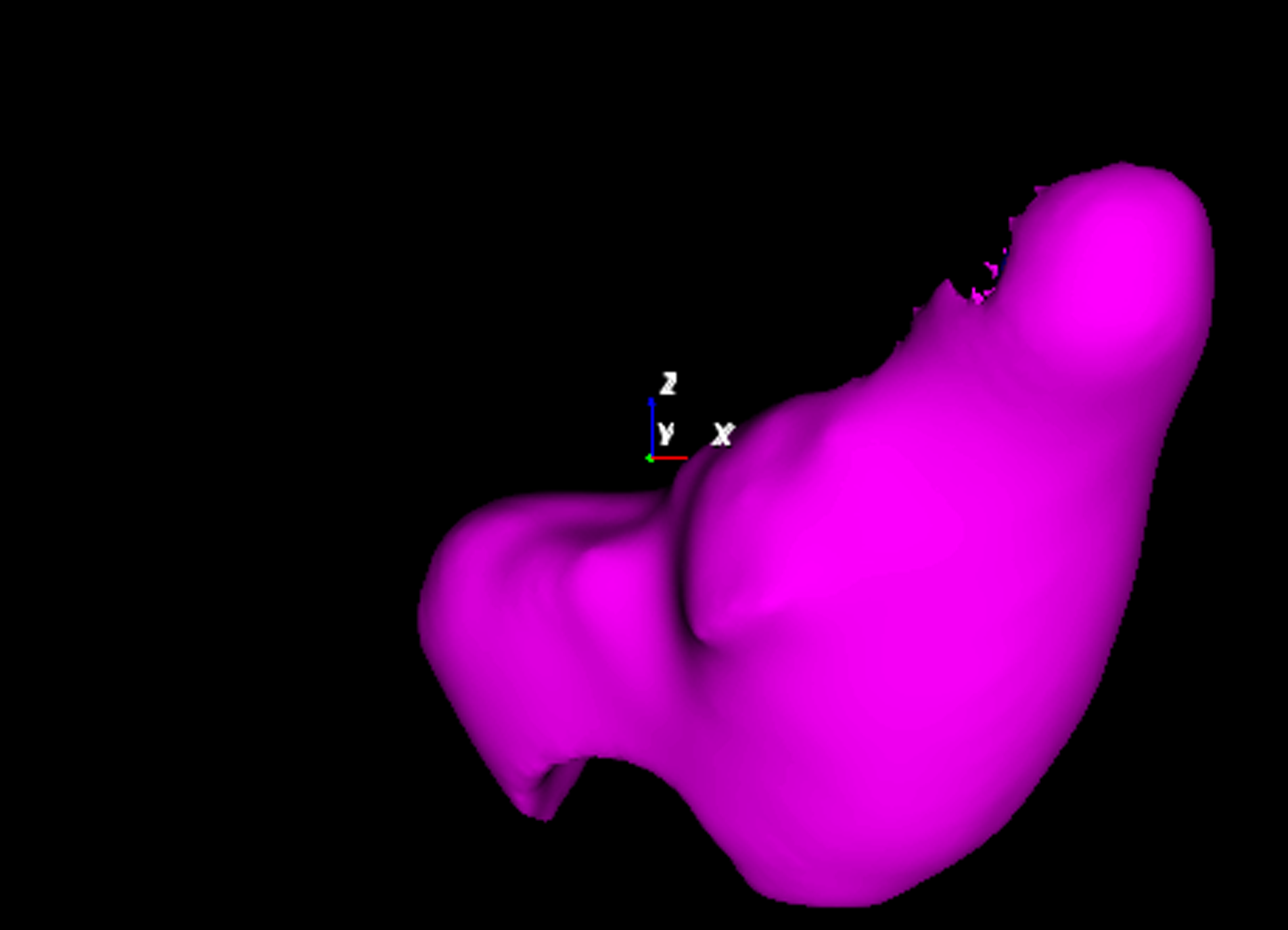

Supplement: Supplementary Movie 2 — 3D image corresponding to Figure 4f, showing the same human carotid artery in vivo, as Movie 1 where PET signal is shown in magenta and CT vascular calcification in blue [file ncomms8495-s3.tif]

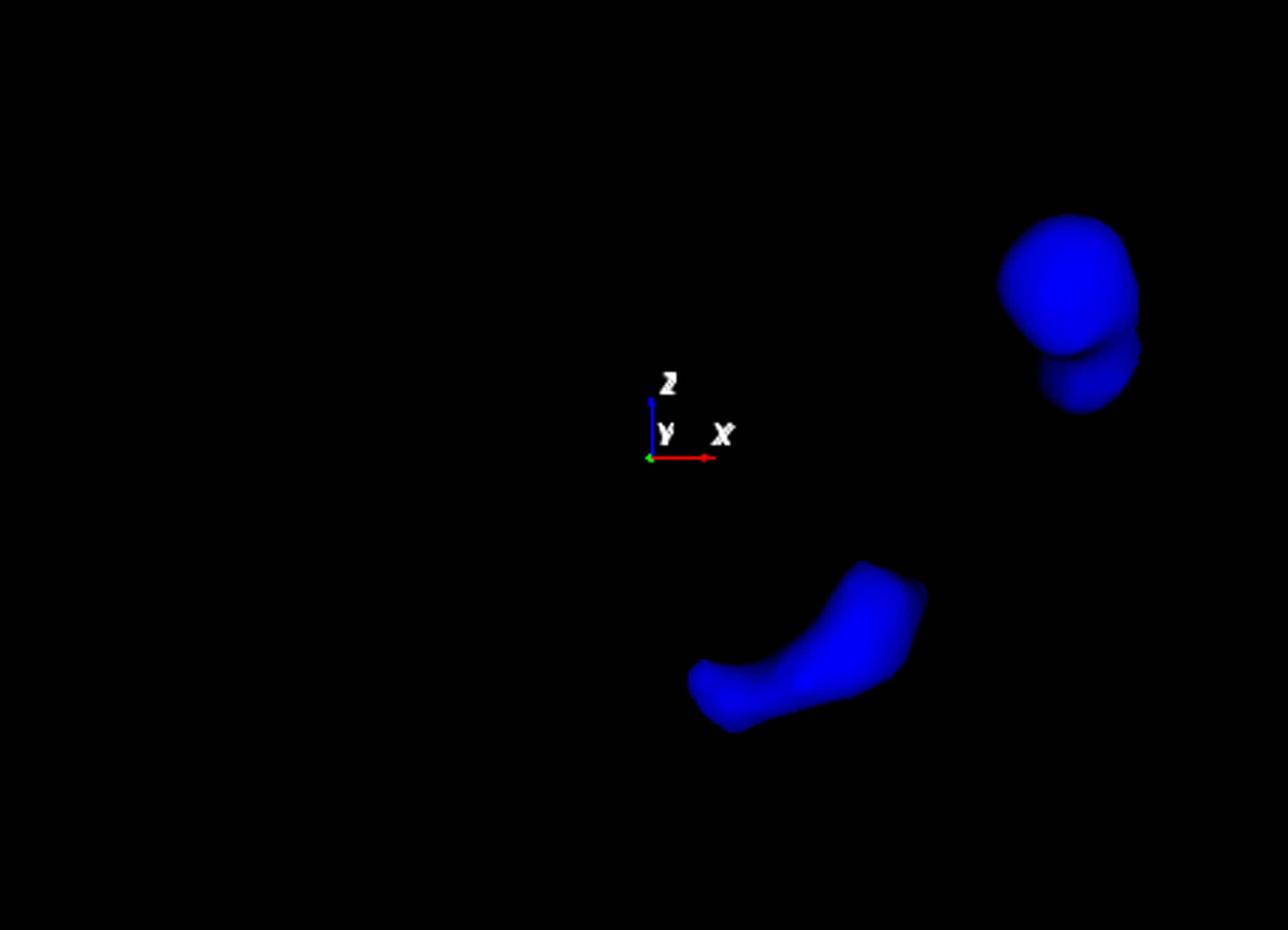

Supplement: Supplementary Movie 3 — 3D image corresponding to Figure 4g showing the same human carotid artery in vivo, as Movie 1 where CT signal is shown in blue [file ncomms8495-s4.tif]

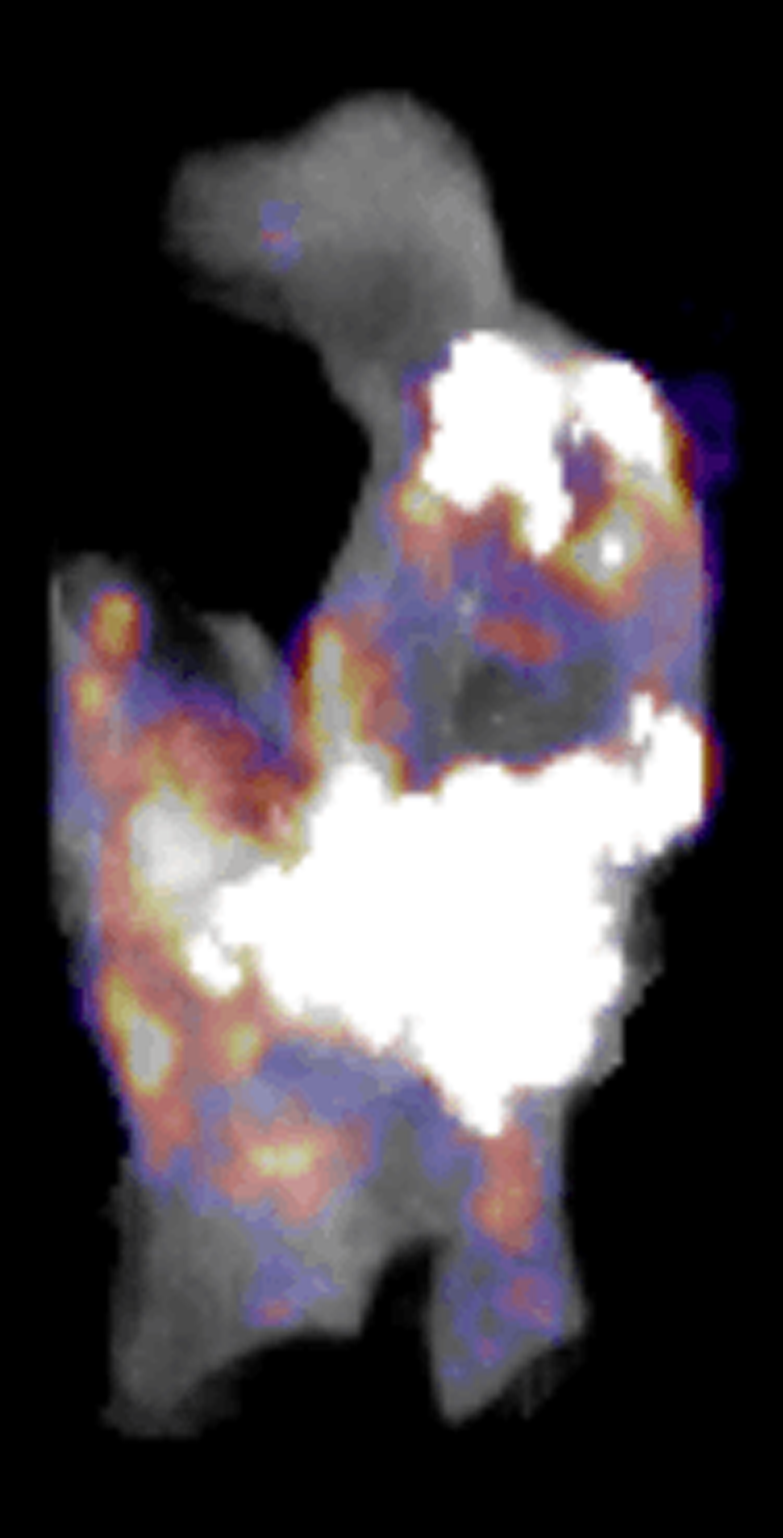

Supplement: Supplementary Movie 4 — 3D image corresponding to Figure 4j showing a human carotid artery ex vivo, following incubation with 18F-sodium fluoride and subsequently imaged using μPET/CT, showing μPET/μCT image without thresholding [file ncomms8495-s5.tif]

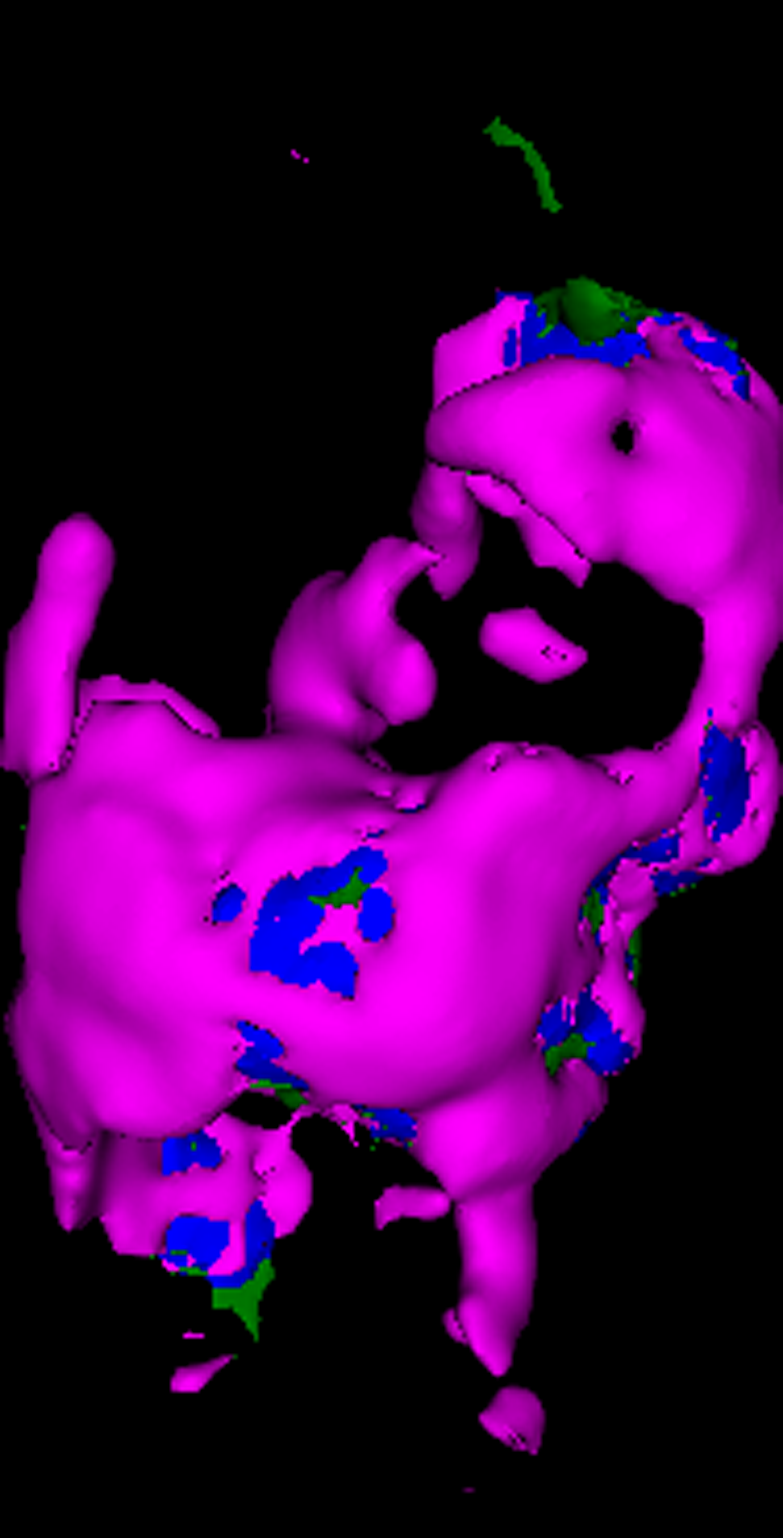

Supplement: Supplementary Movie 5 — 3D image corresponding to Figure 4k showing the same human carotid artery ex vivo as Movie 4, showing 3D thresholded μPET/μCT segments [file ncomms8495-s6.tif]

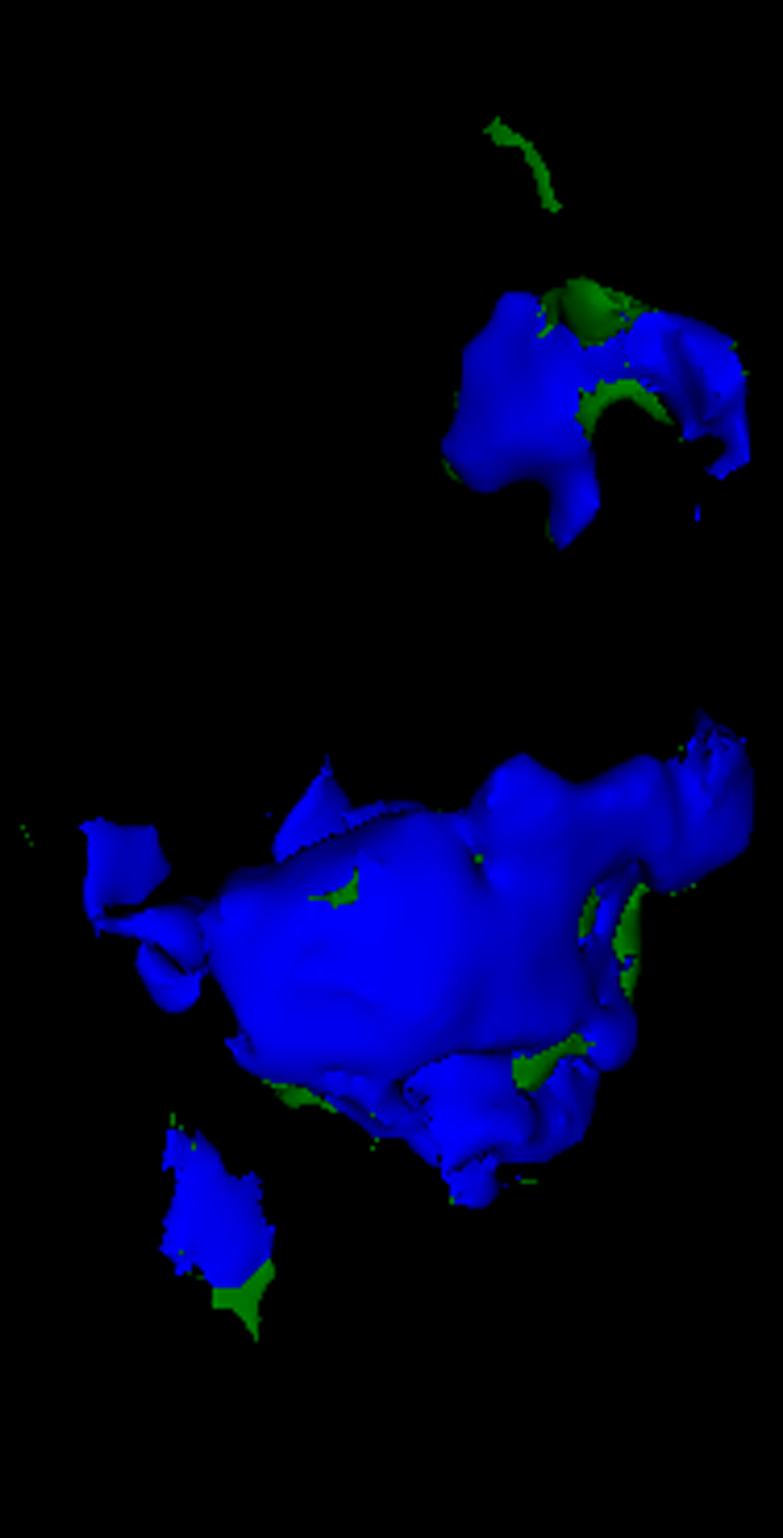

Supplement: Supplementary Movie 6 — 3D image corresponding to Figure 4l of the same human carotid artery ex vivo as Movie 4, showing 3D thresholded μPET/μCT segments [file ncomms8495-s7.tif]

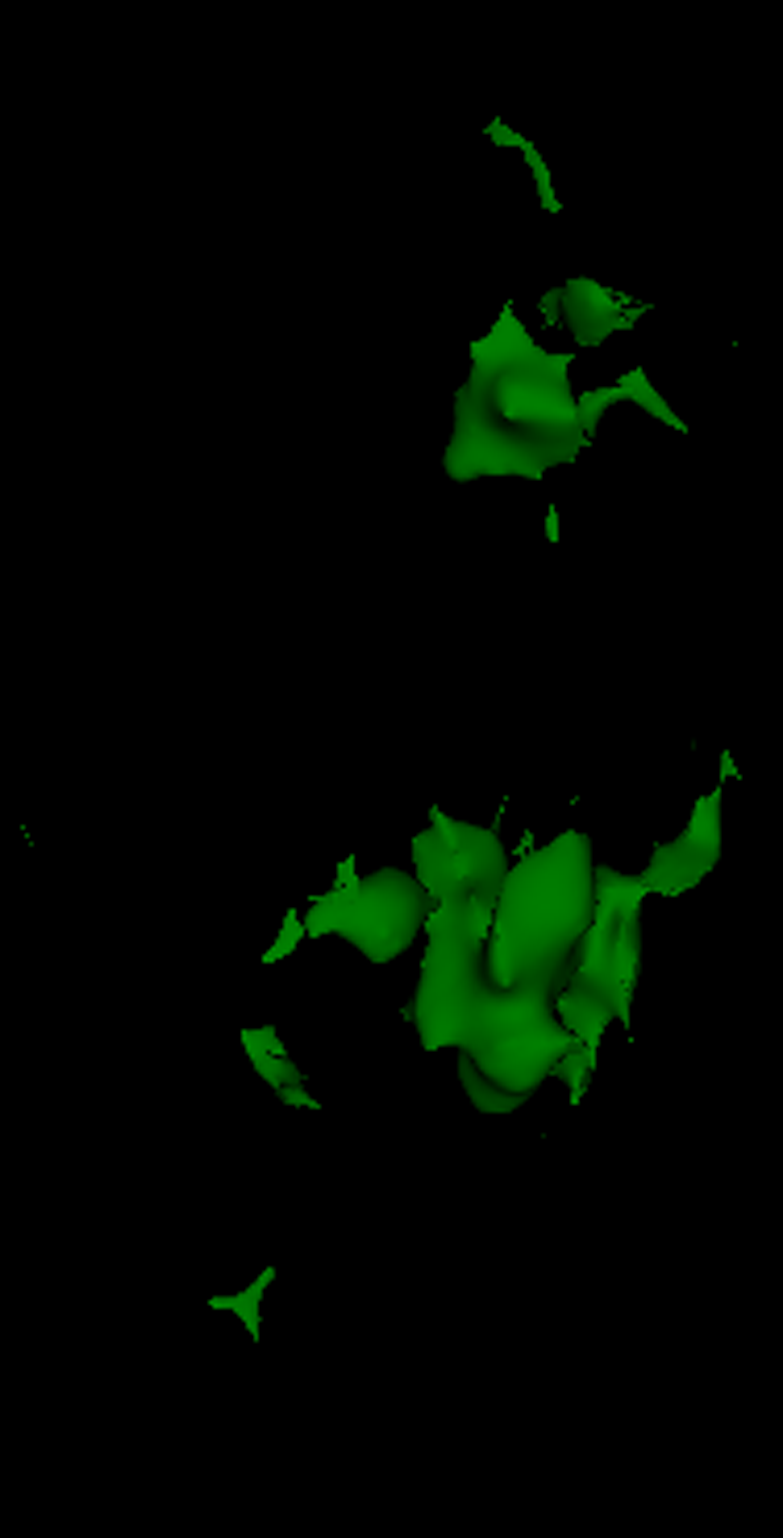

Supplement: Supplementary Movie 7 — 3D image corresponding to Figure 4m of the same human carotid artery as Movie 4, showing 3D thresholded μPET/μCT segments [file ncomms8495-s8.tif]
